# Electrocatalytic CO_2_ reduction to alcohols by modulating the molecular geometry and Cu coordination in bicentric copper complexes

**DOI:** 10.1038/s41467-022-32740-z

**Published:** 2022-08-31

**Authors:** Baiyu Yang, Ling Chen, Songlin Xue, Hao Sun, Kun Feng, Yufeng Chen, Xiang Zhang, Long Xiao, Yongze Qin, Jun Zhong, Zhao Deng, Yan Jiao, Yang Peng

**Affiliations:** 1 Soochow Institute for Energy and Materials Innovations, College of Energy, Key Laboratory of Advanced Carbon Materials and Wearable Energy Technologies of Jiangsu Province, Soochow University, 215006 Suzhou, P. R. China; 2 Jiangsu Key Laboratory of Advanced Negative Carbon Technologies, Soochow Municipal Laboratory for Low Carbon Technologies and Industries, Soochow University, 215123 Suzhou, Jiangsu P. R. China; 3 School of Chemical Engineering and Advanced Materials, The University of Adelaide, Adelaide, SA 5005 Australia; 4 Institute of Functional Nano & Soft Materials (FUNSOM), Jiangsu Key Laboratory for Carbon-Based Functional Materials & Devices, Soochow University, 215123 Suzhou, China

**Keywords:** Electrocatalysis, Metal-organic frameworks, Electrocatalysis

## Abstract

Electrocatalytic reduction of CO_2_ into alcohols of high economic value offers a promising route to realize resourceful CO_2_ utilization. In this study, we choose three model bicentric copper complexes based on the expanded and fluorinated porphyrin structure, but different spatial and coordination geometry, to unravel their structure-property-performance correlation in catalyzing electrochemical CO_2_ reduction reactions. We show that the complexes with higher intramolecular tension and coordination asymmetry manifests a lower electrochemical stability and thus more active Cu centers, which can be reduced during electrolysis to form Cu clusters accompanied by partially-reduced or fragmented ligands. We demonstrate the hybrid structure of Cu cluster and partially reduced O-containing hexaphyrin ligand is highly potent in converting CO_2_ into alcohols, up to 32.5% ethanol and 18.3% *n-*propanol in Faradaic efficiencies that have been rarely reported. More importantly, we uncover an interplay between the inorganic and organic phases to synergistically produce alcohols, of which the intermediates are stabilized by a confined space to afford extra O-Cu bonding. This study underlines the exploitation of structure-dependent electrochemical property to steer the CO_2_ reduction pathway, as well as a potential generic tactic to target alcohol synthesis by constructing organic/inorganic Cu hybrids.

## Abstract (web summary)

Electrocatalytic reduction of CO2 into multi-carbon alcohols of high economic merit offers an effective means to close the carbon cycle. Here the authors show the synergy between inorganic and organic phases derived from rationally designed molecular precursors to produce alcohols in high efficiency.

## Introduction

Electrochemical CO_2_ reduction reactions driven by renewable energy sources to produce value-added chemical fuels and feedstocks offer a promising carbon-negative means to fulfill the carbon neutral goal of mankind^1,2^. Copper and copper-based compounds have been well reckoned highly effective in producing deeply reduced multi-electron products, owning to the suitable intermediates binding energetics that allow serial reaction cascades^3,4^. Among the diverse reaction products including C_1_ and C_2+_ hydrocarbons and oxygenates, ethanol and *n*-propanol, despite their high industrial and economic merits, have been challenging to obtain in high yield and selectivity^5,6^. One possible reason lies in the highly reductive environment at the cathode surface, making the oxygenic intermediates difficult to stay, especially beyond the potentials of C–C coupling. Exquisite catalyst design and fabrication, aiming to tune individual intermediate binding and break the linear scaling relation^7^, are thus needed to produce these multi-carbon alcohols in high selectivity and turnover rates.

To improve the production of oxygenates in CO_2_RR, a few tactics have been practised. First of all, high concentration of local CO has been generally viewed favorable in producing alcohols, including CO added to the feedstock^8,9^ or produced through a tandem catalyst^10,11^. Second, synergies from catalyst and substrate interaction, such as Cu clusters supported on oxidized carbons and metals, have been exploited for effectually boosting the alcohol production^12,13^. Third, nitrogen-doped graphitic carbon and its complexation with Cu moieties have also witnessed considerable oxygenates production^14,15^. Last but not least, the utilization of crystalline defects, especially those are in close proximity, have been recently implemented as a potent strategy to drive alcohol production^16^. In general, the thinking behind these diverse approaches is to lower the overpotential for C–C coupling so as to mitigate the highly reductive electrolytic environment, and the exploitation of a companion active motif to stabilize the oxygenated intermediates^17^. Thus, the development of efficient electrocatalysts based on fundamental understandings of the C–C coupling and oxygenation process is imperative to target alcohol production in CO_2_RR.

Heterogeneous molecular catalysts offer a unique platform for CO_2_RR in that they allow the tuning of intermediates binding by modulating the molecular configuration, and the furnishing of mechanistic understandings via the explicit structure^18,19^. To date, many transition metal-coordinated macrocycle molecules, as well as their complexed forms, have been demonstrated with high CO_2_RR activities to produce majorly C_1_ products^20,21^, possibly owing to their singly isolated metal centers. Bicentric metal complexes, with two metal centers adjacent to each other, are thus highly intriguing, for their possible capabilities in triggering C–C coupling and stabilizing oxygenic intermediates through ligation effects.

In this study, we choose three bicentric Cu complexes based on the expanded and fluorinated porphyrin structure, but different molecular and coordination geometry, to illustrate the structure-property-performance correlation of heterogeneous molecular catalysts in CO_2_RR. The fluorinated compound structure lends all compounds insoluble in aqueous electrolyte. We find the electrochemical stability of the macrocycle molecules are highly dependent on the molecular configuration and coordination sphere, which in turn steers the CO_2_RR pathway. Moreover, we uncover, both experimentally and theoretically, a strong synergy between the in situ generated Cu cluster and partially reduced molecular substrate in concertedly producing oxygenates, with high selectivity of ethanol and *n-*propanol that has been rarely seen in literature.

## Results

### Catalysts synthesis and characterization

**Fig. 1:**
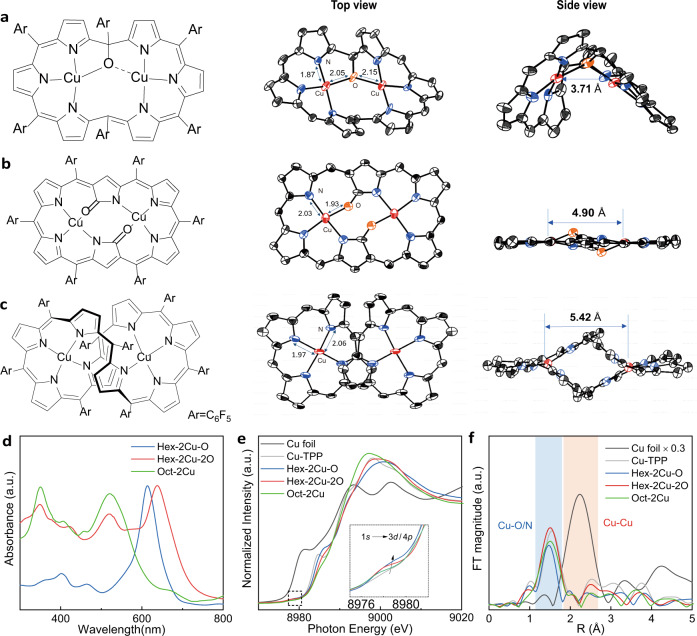
Structure and characterization of the bicentric expanded-porphyrin complexes. **a**–**c** Molecular structure and spatial geometry of Hex-2Cu-O (**a**), Hex-2Cu-2O (**b**), and Oct-2Cu (**c**). **d** UV–Vis spectra of Hex-2Cu-O, Hex-2Cu-2O, and Oct-2Cu. **e** XANES Cu K-edge and **f** FT-EXAFS spectra of Hex-2Cu-O, Hex-2Cu-2O, and Oct-2Cu in reference to Cu-TPP and Cu foil.

Syntheses of the bicentric copper complexes, denoted as Hex-2Cu-O, Hex-2Cu-2O and Oct-2Cu in Fig. 1a–c, respectively, are based on the ring expansion of porphyrins following previous reports (Supplementary Fig. 1)^22–25^. Their chemical structures are confirmed by UV–Vis (Fig. 1d) and Mass spectroscopy (Supplementary Figs. 2–4), matching well with the literature results^23,24^ and theoretical values. For Hex-2Cu-O, each of the two copper ions is bound to one bridging meso-oxygen atom and three pyrrolic nitrogen atoms in a distorted quadrilateral configuration. The central Cu–O–Cu bonding fights against the tension of the expanded porphyrin, resulting in a gabled structure with a Cu–Cu distance of 3.7 Å (Fig. 1a). In Hex-2Cu-2O, the doubly N-confused hexaphyrin provides two carbonyl oxygens to allow both copper ions to be independently anchored by the surrounding three N and one O atoms, also in a square-planar geometry but with milder Cu-O strain. As a result, the overall structure of the macrocycle is planar with a Cu–Cu distance of 4.9 Å (Fig. 1b). Oct-2Cu has a C_2_ symmetry with the two copper atoms bound to two staggered porphyrin rings (Fig. 1c), each in the form of a hemi-macrocycle with central Cu−N_4_ coordination, and in this case the bicentric Cu−Cu distance is 5.4 Å.

X-ray absorption near edge structure (XANES) spectra in Fig. 1e reveal that all the Cu oxidation states in Hex-2Cu-O, Hex-2Cu-2O and Oct-2Cu are Cu^2+^, according to their absorption edges and white-line intensities in reference to Cu foil and copper tetraphenylporphyrin (Cu-TPP)^26^. A close inspection on the pre-edge absorption at ca. 8978 eV (1 *s*→3*d*/4*p*) discerns stronger peak intensities of Hex-2Cu-O and Hex-2Cu-2O when compared to Oct-2Cu and Cu-TPP (Fig. 1e inset), attesting to the deviated quadrilateral Cu–N_3_O moieties with reduced coordination symmetry^27^. Among the three bicentric complexes, Oct-2Cu has the lowest pre-edge absorption, but still higher than that of Cu-TPP. This is owing to the out-of-plane Cu–N_4_ coordination in Oct-2Cu in comparison to the perfect D_4h_ symmetry of Cu-TPP. Fourier-transform extended X-ray adsorption fine structure (FT-EXAFS) spectra in Fig. 1f indicates the average length of the first shell Cu-O/N bonding in Hex-2Cu-O is slightly shorter than those of Hex-2Cu-2O and Oct-2Cu, owing to the fact that Cu atoms in Hex-2Cu-O are more strongly coordinated to the nitrogen atoms but weakly associated with the shared oxygen (see Supplementary Table 1 for crystallographic bond-length data). The shortened Cu–N bonding has been also witnessed for under-coordinated Ni-N_3_ centers in nickel phthalocyanine^28^. By contrast, the contribution of oxygen to the standalone Cu–N_3_O coordination in Hex-2Cu-2O is more eminent and indicative of a stronger ligand field. As for Oct-2Cu, the conjugated porphyrin ligands in the staggered configuration confronts the Cu–N bonds (Fig. 1c, side view), making the average Cu–N bond length slightly shorter than that of Cu-TPP. Collectively from these observations, it can be inferred that a more intensive intramolecular tension exists in Hex-2Cu-O and Oct-2Cu, jeopardizing their thermodynamic and electrochemical stability.

### Electrochemical CO_2_RR performances

Electrocatalytic CO_2_RR performances of Hex-2Cu-O, Hex-2Cu-2O and Oct-2Cu loaded onto Ketjen black (KB) were first evaluated in a gas-tight H-cell containing CO_2_-saturated 0.1 M KHCO_3_ as the electrolyte. Linear sweep voltammetries (LSVs) show that the overall current densities of Hex-2Cu-O and Oct-2Cu in the CO_2_-saturated electrolyte are higher than those in Ar-saturated electrolyte, whereas Hex-2Cu-2O exhibits similar current densities in both CO_2_ and Ar-saturated electrolytes (Supplementary Fig. 5). This suggests the electron transfer number in CO_2_RR catalyzed by Hex-2Cu-2O is close to that in HER, yielding the majority of 2e^−^ reduction products such as H_2_, CO or HCOOH (assuming the same type/number of active sites were engaged). In this vein, there should be more electron transfer involved in CO_2_RR catalyzed by Hex-2Cu-O and Oct-2Cu, resulting in more deeply reduced products of >2e^−^. In addition, all the compound catalysts show significantly higher LSV current density than that of the KB alone in CO_2_-saturated electrolyte, confirming that KB, as a conductive agent, does not affect the performance evaluation of the catalysts.

**Fig. 2:**
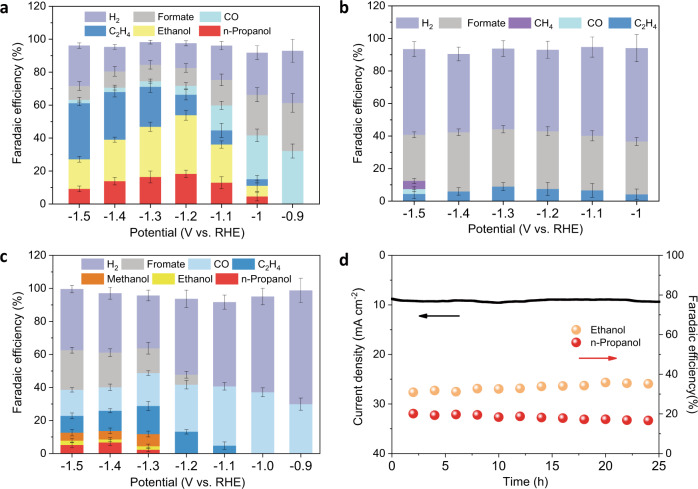
Electrocatalytic CO_2_RR performances. **a**–**c** FEs of various reduction products at different potential for Hex-2Cu-O (**a**), Hex-2Cu-2O (**b**), and Oct-2Cu (**c**). **d** Chronoamperometric *i–t* curve at −1.2 V for Hex-2Cu-O with the evolution of ethanol and *n-*propanol FEs. Error bars represent the standard deviation of three independent measurements.

In the controlled-potential electrolysis (CPE) from –0.9 to –1.5 V (vs RHE without iR correction, all potentials are referenced to this format hereafter unless specifically noted), Hex-2Cu-O produces mainly CO and formate at low overpotentials (Fig. 2a). Starting from −1.1 V and beyond, C_2+_ products including ethylene, ethanol and *n-*propanol constitute the main CO_2_RR products. In particular, at −1.2 V the total Faradaic efficiency (FE) of multi-carbon alcohols increases to 50.8% (ethanol: 32.5%; *n-*propanol: 18.3%), which has been rarely seen in the literature (Supplementary Table 2). At −1.3 V, the total FE of C_2+_ products reaches to its maximum of 71% (Supplementary Fig. 6). Afterwards, both the FEs of ethanol and *n-*propanol decreases, whereas the FE and partial current density of ethylene continually rise (Supplementary Fig. 7). At −1.5 V, the FE of ethylene increases to 34%, surpassing the alcohols with a total FE of 27%. These observations support the common view that the yield of oxygenates, compared to their hydrocarbon counterparts, is favored at relatively higher potentials. In contrast, Hex-2Cu-2O produces majorly hydrogen and formate during the whole CPE test, apart from a small amount of ethylene as well as trivial methane at −1.5 V (Fig. 2b). Oct-2Cu yields mainly CO and hydrogen when a relatively low bias of −0.9 or −1.0 V is applied (Fig. 2c). Starting from −1.1 V, C_2_H_4_ starts to emerge and reaches a maximum FE of 17% at −1.3 V. At this point, alcohols are also detected in the electrolyte, but the FEs are significantly lower than those attained by Hex-2Cu-O. Note that methanol accounts for a significant portion of the alcohols catalyzed by Oct-2Cu, which was not observed for Hex-2Cu-O. When KB alone is used for electrolysis, the CO_2_RR product is dominated by hydrogen (Supplementary Fig. 8).

Chronoamperometric *i–t* test was further carried out to examine the electrocatalytic stability of Hex-2Cu-O at −1.2 V with the liquid products collected intermittently for ethanol and *n-*propanol analysis by NMR. Supplementary Fig. 9 exemplifies the ^1^H-NMR spectra of the products from Hex-2Cu-O after 20 h of test in CO_2_ or Ar-saturated electrolyte. With CO_2_ purged into the electrolyte, prominent methyl hydrogen peaks of ethanol and *n-*propanol (with a real-time total FE of 53.1%) are observed in the NMR spectrum at δ = 1.0 and 0.75, respectively (Supplementary Fig. 9a). When CO_2_ is replaced with Ar, no hydrocarbons can be identified except for DMSO used as the internal standard (Supplementary Fig. 9b), indicating that the produced alcohols are solely converted from CO_2_. During the entire CO_2_RR testing period of 25 h, the total current density maintained nearly constant at ∼9.4 mA cm^−2^, and the FEs of ethanol and *n-*propanol were well retained at 35.3 and 16.7%, respectively, by the end of the test (Fig. 2d and Supplementary Table 3). Such a high alcohol selectivity and electrocatalytic stability of Hex-2Cu-O intrigues us to interrogate its structural stability or mutability during the electrolytic process in contrast to Hex-2Cu-2O and Oct-2Cu.

### Structural evolution of the catalysts during CO_2_RR

Matrix-assisted laser desorption ionization-time of flight (MALDI-TOF) mass spectrometry (MS) taken on Hex-2Cu-O after 10 h of CPE at −1.2 V reveals a series of peaks of various m/z ratios (Supplementary Fig. 10), in stark contrast to the localized peaks at *m/z* = 1601.9 for pristine Hex-2Cu-O (Supplementary Fig. 2). While the peaks centered at *m/z* = 1462.5 correspond to free-base hexaphyrin, those centered at *m/z* = 1541.4 can be ascribed to partially reduced Hex-2Cu-O with one Cu atom stripped away. Therefore, the post-electrolytic Hex-2Cu-O should contain a mixture of free-base, mono-, and bicentric hexaphyrins, as well as possible Cu^0^ moieties reduced out from the complexes (which will be confirmed later). By contrast, post-electrolytic Hex-2Cu-2O shows an unchanged peak at *m/z* = 1615.7 (Supplementary Fig. 11 vs Supplementary Fig. 3), suggesting that the original molecular structure of Hex-2Cu-2O was kept intact during electrolysis. Thus, one can conclude that under the same electrolytic conditions Hex-2Cu-O is less electrochemically stable than Hex-2Cu-2O, and hence the CO_2_RR activity of the former must have come from its mutated structure. For Oct-2Cu, post-electrolytic MS did not detect any signals from the original macrocycles (i.e. Oct-2Cu or Octaphyrin) other than a bunch of fragments below *m/z* = 1100 (Supplementary Fig. 12). This indicates the conjugated octaphyrin ring of Oct-2Cu can be fully ruptured during electrolysis.

The structural mutation of Hex-2Cu-O was also witnessed by UV–vis spectroscopy. After 10 h of electrolysis at −1.2 V, post-electrolytic Hex-2Cu-O was dissolved in deoxygenated ethanol and subjected to UV–vis characterization. Compared to the spectrum of pristine Hex-2Cu-O, that of the post-electrolytic Hex-2Cu-O exhibits two new absorptions at 602 and 741 nm, which are also observed on the chemically reduced Hex-2Cu-O by reacting with NaBH_4_ (Supplementary Fig. 13). In general, the UV–vis spectrum of post-electrolytic Hex-2Cu-O contains signatures from both the pristine and chemically reduced Hex-2Cu-O, confirming that Cu centers in Hex-2Cu-O are partially stripped. For comparison, the UV–Vis spectrum of post-electrolytic Hex-2Cu-2O remains unchanged after the same 10-h electrolysis at −1.2 V (Supplementary Fig. 14), which echoes previous observation from mass spectrometry. The UV–Vis spectrum of post-electrolytic Oct-2Cu shows only a few weak absorptions upon losing most of the original octaphyrin signatures, providing further evidence for the cleavage of the Oct-2Cu macrocycles (Supplementary Fig. 15).

X-ray photoelectron spectroscopy (XPS) taken on Hex-2Cu-O before and after 10 h of electrolysis exhibits drastically weakened N-Cu bonding at 399.2 eV in the high-resolution N 1*s* spectra (Supplementary Fig. 16), corroborating the stripping of Cu from the complex. In the pristine Hex-2Cu-O, the Cu 2*p* signals contain a majority of Cu^2+^ state at 935.4 (2*p*^3/2^) and 955.3 (2*p*^1/2^) eV (Supplementary Fig. 17a). After electrolysis, despite of the overall low signal intensity, both Cu^2+^ and Cu^+^/Cu^0^ peaks are discernable (Supplementary Fig. 17b). The N 1 *s* spectra of Oct-2Cu before and after electrolysis display a similar trend to that of Hex-2Cu-O, indicating that Cu^2+^ in the molecule was also reduced to Cu^+^/Cu^0^ (Supplementary Fig. 18). No Cu^2+^ state was observed in the Cu 2*p* spectrum of Oct-2Cu after electrocatalysis (Supplementary Fig. 19). Further in the Auger Cu LMM spectra, post-electrolytic Hex-2Cu-O features a broad peak that can be decoupled to coordinated Cu^2+^ (572.2 eV) and superficially oxidized Cu^δ+^ (569.8 eV) species, whereas post-electrolytic Oct-2Cu shows majorly the superficial Cu^δ+^ apart from a small shoulder of Cu^0^ (Supplementary Fig. 20). This corroborates the view that Oct-2Cu can be fully reduced during electrolysis. In stark contrast, both the XPS N 1*s* and Cu 2*p* spectra of Hex-2Cu-2O before and after electrolysis remain mostly unchanged (Supplementary Figs. 21 and 22). Consequently, our XPS analyses on the three compound catalysts before and after electrolysis further attest to the good electrochemical stability of Hex-2Cu-2O, the easily fragmented Oct-2Cu, and the partially reduced Hex-2Cu-O.

**Fig. 3:**
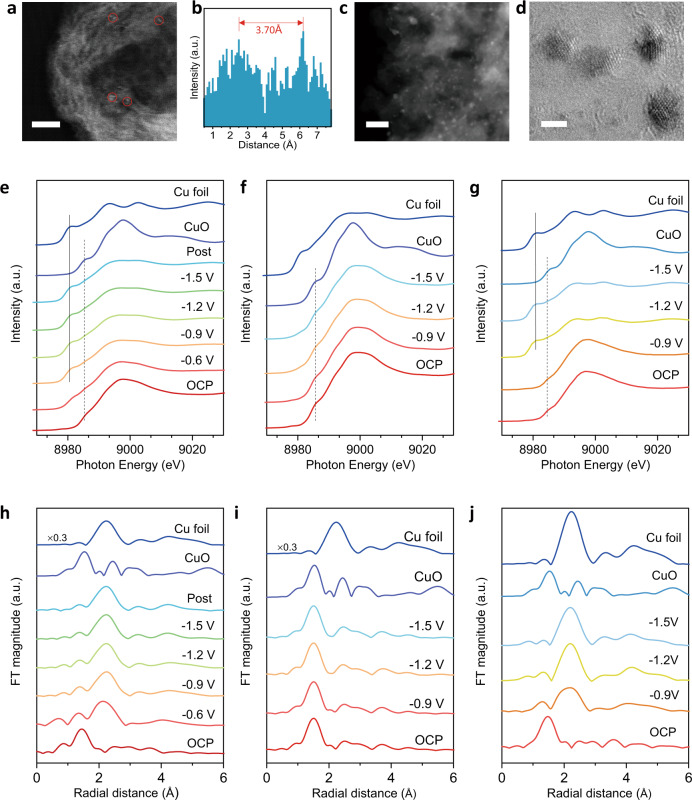
Ex-situ TEM images and operando XAS. **a** Representative HAADF–STEM images of Hex-2Cu-O/KB (1:10 dilution) showing the presence of isolated Cu pairs marked by red circles. **b** Line-scan intensity profile drawn upon one of the diatomic Cu pairs. **c**, **d** High-resolution TEM (**c**) and Cs-TEM (**d**) images of Hex-2Cu-O/KB after 10 h of electrolysis at –1.2 V. Scale bars, 2 nm (**a**), 50 nm (**c**) and 2 nm (**d**). **e**–**j** In situ XAS measurements under electrocatalytic CO_2_RR conditions: Cu K-edge XANES spectra (**e**–**g**) and Fourier-transform Cu K-edge EXAFS spectra (**h**–**j**) for Hex-2Cu-O, Hex-2Cu-2O and Oct-2Cu, respectively.

To visualize the Hex-2Cu-O complex loaded onto KB before and after electrolysis, high-angle annular dark-field and aberration-corrected scanning transmission electron microscopy (HAADF-STEM) was employed. Figure 3a confirms the bicentric configuration of pristine Hex-2Cu-O, revealing isolated diatomic Cu pairs dispersed onto the carbon support. The corresponding line-scan profile displays a bimodal spacing of ca. 3.7 Å (Fig. 3b), closely matching the theoretical Cu–Cu spacing in the molecular structure shown in Fig. 1a. After CO_2_RR test for 10 h, organic solvents were used to wash away the organic phase on the electrode surface, on which TEM discloses numerous copper clusters of several nanometers (Fig. 3c). The atomic arrangement of the Cu clusters is better clarified by spherical aberration-corrected TEM (Cs-TEM), revealing multi-facet exposure and highly under-coordinated edge atoms (Fig. 3d) which have been well reckoned as active sites for *CO adsorption and C–C coupling in CO_2_RR^13,26,29^. Additionally, no distinct electron paramagnetic resonance (EPR) signal was observed for the pristine Hex-2Cu-O due to strong anti-ferromagnetic exchange between the two Cu^2+^ ions (Supplementary Fig. 23). Post-electrolytic Hex-2Cu-O, however, exhibits typical EPR spectrum of Cu^II^ porphyrin (with *S* = 1/2) as a result of ligand hydrogenation^23^. The high-resolution TEM images, together with the comprehensive spectroscopic evidences above, unanimously testify the structural mutation of Hex-2Cu-O during electrolysis to form a hybrid structure composed of Cu clusters and partially reduced Hex-2Cu-O.

Furthermore, to monitor the Cu evolution in both Hex-2Cu-O, Hex-2Cu-2O and Oct-2Cu during CO_2_RR, in situ XAS measurements were carried out. For Hex-2Cu-O, the serial XANES spectra recorded by ramping down the applied potential from −0.6 to −1.5 V unveil the decay of Cu^2+^ at 8984 eV but emergence of Cu^0^ at 8980 eV even at low overpotentials (Fig. 3e)^30,31^. In the corresponding FT-EXAFS spectra, prominent Cu–Cu bonding at *R* = 2.2 Å can be observed at all applied potentials (Fig. 3h). Fitting the spectrum at −1.2 V to the FCC model of metallic Cu results in a Cu–Cu coordination number (CN) of 4.9 (Supplementary Fig. 24, Table 4), underlining its small cluster size and highly under-coordinated nature that correspond to the Cs-TEM observation. The Cu–N/O coordination at *R* = 1.5 Å is persistent throughout the whole testing period, confirming that not all Cu centers are stripped off from the complex^32^. This is in line with the MS, UV–Vis, and XPS observations that collectively point to the coexistence of both metallic Cu species and incompletely reduced Hex-2Cu-O complexes under the CO_2_RR condition. Note that the Cu reduction in Hex-2Cu-O is not reversible, as evidenced by the Cu^0^ state and Cu–Cu bonding retained in the post-electrolytic sample (Fig. 3h). In stark contrast, both the in situ XANES and FT-EXAFS spectra taken on Hex-2Cu-2O exhibit minimal change of Cu oxidation and coordination states from −0.9 to −1.5 V (Fig. 3f, i), evidencing, once again, its electrochemical stability during CO_2_RR. The in situ XAS spectra of Oct-2Cu also testify the reduction of Cu^2+^ to Cu^0^ under negative bias (Fig. 3g, j). At −1.5 V, both the spectral features of XANES and FT-EXAFS well match those of the copper foil without discernable Cu–N signals, further supporting the view that Cu clusters dominate the post-electrolytic Cu species, which coincides earlier XPS observations.

Similar structural evolution of Hex-2Cu-O with the emergence of Cu clusters during CO_2_RR was also witnessed by Time-lapse XAFS at a constant potential of −1.2 V (Supplementary Fig. 25). In just 8 min, Cu centers in the complex were already partially reduced, as witnessed by the coexistence of Cu^0^ and Cu–N/O species in both the XANES and FT-EXAFS spectra. After 24 min, the whole catalyst system was stabilized, with similar Cu oxidation and coordination states till the end of the test. It is thus surmised that metallic Cu reduced from organometallic precursors might impose self-constraint on particle growth due to the ligand tethering effect and catalyst-support interaction, which have been demonstrated in previous literature reports^4,33,34^, and further confirmed here by plotting the potential-dependent Cu–Cu CNs revealing the slow-down of particle growth with increasing bias (Supplementary Fig. 26). Collectively, our comprehensive characterizations above should help to rationalize the great electrocatalytic activity and stability of Hex-2Cu-O as witnessed in Fig. 2d.

### Mechanistic insights into the high C_2+_ and alcohol production

Our choice of Hex-2Cu-O, Hex-2Cu-2O and Oct-2Cu as the CO_2_RR catalysts offers a paradigm for illustrating the correlation of structure-property-performance. For the two bicentric hexaphyrin complexes, although all copper ions are coordinated to three nitrogen and one oxygen, the geometry and symmetry of the coordination spheres are quite different. The shared oxygen atom in Hex-2Cu-O and the intramolecular tension from the gabled structure make the complex less thermodynamically stable, and thus the Cu atoms are more active and can be stripped off to form metal clusters. Comparatively, the quasi-square planar configuration of Cu–N_3_O in Hex-2Cu-2O with a stronger ligand field is more stable, resilient even to high negative bias. As for Oct-2Cu, the staggered porphyrin ligands that also create intramolecular tension make it both thermodynamically and electrochemically less stable and could fall apart under negative potentials. These arguments are well supported by cyclic voltammetry (CV) taken in Ar-saturated electrolyte (Supplementary Figs. 27 and 28). Hex-2Cu-2O shows a similar voltammogram to that of free-base hexaphyrin without notable Cu valence change in the potential range between −0.8 and 1.2 V (Supplementary Fig. 27), attesting to its stable configuration. Both Hex-2Cu-O and Oct-2Cu exhibit prominent redox features on both cathodic and anodic scans, in accordance with the Cu valence change observed by in situ XAFS (Supplementary Fig. 28). Specifically, the cathodic peak at −0.39 V for Hex-2Cu-O can be ascribed to the reduction Cu^2+^ to Cu^+^/Cu^0^, as confirmed by the ex-situ XPS Cu 2*p* spectra taken at different bias (Supplementary Fig. 29). The other two cathodic peaks at −0.03 and 0.36 V are not associated with Cu valence change, but possibly related to the ligand redox behavior^35^. For Oct-2Cu, the ligand reduction/fragmentation is mingled with Cu valence change, and so that a broad hump is observed on the cathodic scan. Additionally, all the anodic peaks at potentials higher than 0.5 V indicate the re-oxidation of Cu from its reduced form. Thus, for both Hex-2Cu-O and Oct-2Cu the actual CO_2_RR activity should be originated from a hybrid structure composed of Cu clusters and reduced ligands. Then, the difference between Hex-2Cu-O and Oct-2Cu is that the ligand of the former contains oxygen and is partially reduced, whereas that of the latter contains no oxygen and is fully fragmented. Based on this cognition, we next proceed to unravel how such a complex structure of partially reduced Hex-2Cu-O work to produce alcohols as the major product in CO_2_RR.

**Fig. 4:**
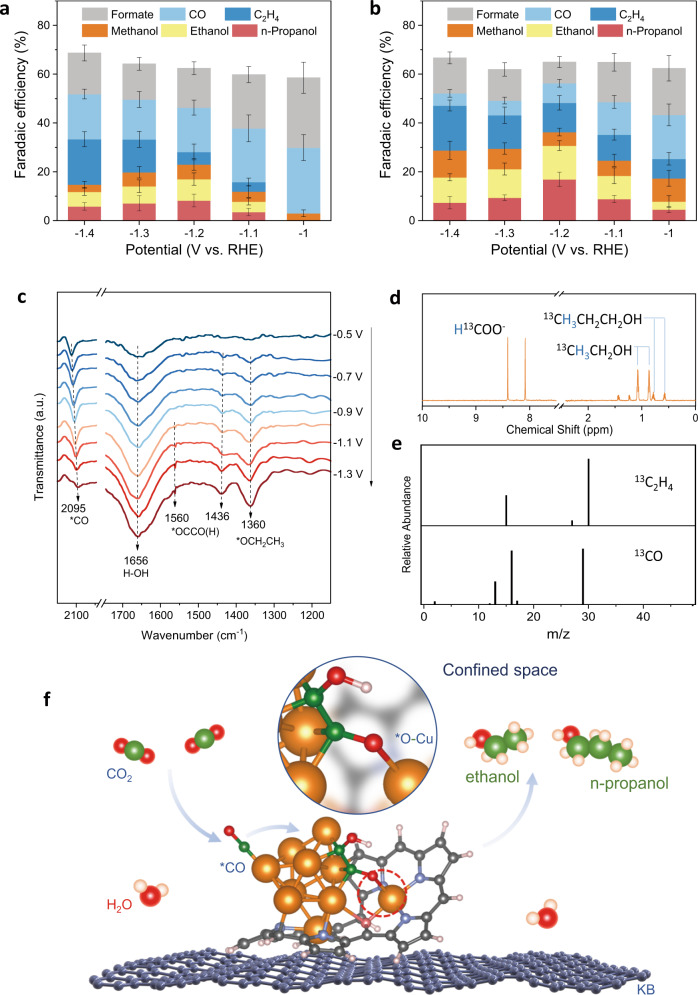
Mechanistic investigations into the alcohol production by Hex-2Cu-O. **a**, **b** CO_2_RR product distribution and the corresponding FEs at different potential for **a** Cu clusters remained on the electrode after ethanol soaking to remove the organic phase and **b** the mixture of Cu-clusters/Hexaphyrins after casting back the solute. **c** Operando ATR-SEIRAS spectra taken by ramping down the applied potential from −0.5 to −1.3 V in CO_2_-saturated 0.1 M KHCO_3_. **d** ^1^H-NMR spectrum of the liquid products and **e** GC-MS-spectra of the gas products produced by isotopic ^13^CO_2_RR at −1.2 V. **f** The proposed C–C coupling and oxygenation mechanism for producing alcohols on restructured Hex-2Cu-O. color codes: Cu, orange; C, gray (Hex-2Cu-O), navy (KB) and green (reaction intermediates); O, magenta (Hex-2Cu-O) and red (reaction intermediates); H, pink (Hex-2Cu-O). Error bars represent the standard deviation of three independent measurements.

To elucidate the synergy from the Cu clusters and partially reduced Hex-2Cu-O complexes in yielding multi-carbon products, we soaked the Hex-2Cu-O electrode in ethanol to remove the organic phase after CO_2_RR at −1.2 V. As a result, the catalyst remained on the electrode should comprise majorly Cu clusters supported on KB, as evidenced by EDX images before and after soaking (Supplementary Figs. 30, 31). Elemental analysis further shows that the Cu clusters remained on the electrode account for ∼82% of the original Cu content, and that the N content is notably reduced after soaking (Supplementary Table 5). In the subsequent retesting of CO_2_RR, the FE of multi-carbon products at −1.2 V decreases from 66.2% for the original catalyst to 22.1%, in which ethanol and *n-*propanol account for only 8.8% and 8.2%, respectively. Meanwhile, the total efficiency of C_1_ products (including formate, CO and methanol) increases from 16.3% to 40.3% (Supplementary Fig. 32). This result is somehow similar to the product distribution observed on Oct-2Cu, which forms copper clusters and fragmented ligands during electrolysis. Collectively, these observations help to justify the role of the partially reduced Hex-2Cu-O in contributing to both C_2+_ and alcohol production. To reinforce this point of view, we further casted the solute from the soaking solution back onto the electrode (Supplementary Fig. 33) and observed that the alcohol production was partially restored. Compared to the ethanol-soaked electrode, the regenerated hybrid catalyst comprising the cast-back organic phase and Cu clusters is able to augment the FEs of oxygenates and hydrocarbons at all applied potentials from −1.0 to −1.4 V (Fig. 4a, b). These post-mortem treatments strongly support the interplay between Cu clusters and partially reduced Hex-2Cu-O ligands to synergistically promote C_2+_ alcohol production.

In situ attenuated total reflection surface-enhanced infrared absorption spectroscopy (ATR-SEIRAS) was employed to probe the intermediates associated with multi-carbon alcohol production on Hex-2Cu-O (Fig. 4c). A prominent absorption band at 2095 cm^−1^, attributed to the C ≡ O stretching mode of *CO^36^, is observed at all potentials from −0.5 to −1.3 V, and its intensity decreases with the increasing bias. This suggests that at negative potentials more CO attend to the C–C coupling reactions to form multi-carbon products, which have been reported in many previous studies^16,37^. Meanwhile, the peak shows a red shift with increasing bias, which is ascribed to the Stark shift caused by strong electronic interaction between *CO and the under-coordinated metal cluster^38^. The peaks located at 1560 cm^−1^ and 1436 cm^−1^ growing conspicuously with decreasing potential can be designated to the C-O vibration of adsorbed *OC–CO(H), a strong evidence for the direct CO-CO dimerization on Cu clusters^39^. As reported in previous studies^13,16^, one of the most probable rate-limiting steps for generating C_2_ intermediates is the *CO dimerization, which is greatly facilitated by concentrated CO adsorption on under-coordinated Cu sites. More importantly, the growing peak at 1360 cm^−1^ is ascribed to the stretching of surface-bound *OCH_2_CH_3_ species^40^, which is considered a key intermediate in the C_2+_ pathway to form alcohols. Note that the premature formation of intermediates before the actual show-up of the corresponding products has been often seen in literature and attributed to their strong surface absorption prior to an endothermic release step^40,41^.

To further confirm the carbon source of the produced oxygenates is indeed from the CO_2_ input, CPE measurements using ^13^CO_2_ as the feedstock were carried out. ^1^H-NMR spectrum acquired on the liquid products reveals doublets at 0.7 and 1 ppm, respectively corresponding to the ^13^C-labeled *n-*propanol and ethanol (Fig. 4d). Meanwhile, GC-MS spectrum of the gas products exhibits signals of *m/z* = 30 and *m/z* = 29 attributed to ^13^C_2_H_4_ and ^13^CO, respectively, further affirming ^13^C as the sole carbon source in all CO_2_RR products (Fig. 4e).

### DFT modeling

In the existing reports of organometallics-derived copper catalysts, metallic copper has been often perceived as the only motif responsible for CO_2_ reduction^42,43^. However, in the current comparative study both the characterization results and control experiments suggest there exists a synergy between the Cu clusters and partially reduced O-containing hexaphyrin complexes in promoting C_2+_ production, especially the alcohols. Although the exact structure of this hybrid complex is difficult to characterize explicitly, we still build a structure model, based on all the experimental evidence gained above as well as the best scientific guess, for the restructured Hex-2Cu-O by placing a Cu cluster adjacent to the partially reduced Hex-2Cu-O molecule containing only one coordinated Cu atom. This results in a confined space between the agglomerated Cu cluster and unreduced single Cu center in the vicinity. In the following DFT investigations based on this model (Fig. 4f), we will show the resultant unique coordination environment (i.e., the confined space affording an extra O–Cu bond) would significantly promote C–C coupling by breaking the scaling relationship between key intermediates and enhance alcohol selectivity by reserving oxygen in the hydroxyl group^44^.

All the constructed models are listed in Supplementary Fig. 34. After geometry optimization, the structure of Hex-2Cu-O bends to a curved shape, coinciding with the molecular structure determined by X-ray crystallography^23,24^. The hybrid structure of Cu cluster and partially reduced Hex-2Cu-O is represented by a nine Cu atom cluster (Cu_9_) immobilized on the Cu-reduction-induced vacancy on Hex-2Cu-O, and denoted as R-Hex-2Cu-O. The curved structure of Hex-2Cu-O forms a confined space between the Cu_9_ cluster and the adjacent single Cu center, which are ~3.84 Å apart (Supplementary Fig. 34). This model is reasonable considering the similar size of the Cu cluster to that of the hexaphyrin molecule (Fig. 3d). Furthermore, to include the impact of carbon-based conducting agent Ketjen Black (KB) in the experiment, a 5 × 5 × 1 graphene substrate was introduced to support the hybrid structure (R-Hex-2Cu-O/G), which was used for all reaction mechanism study.

**Fig. 5:**
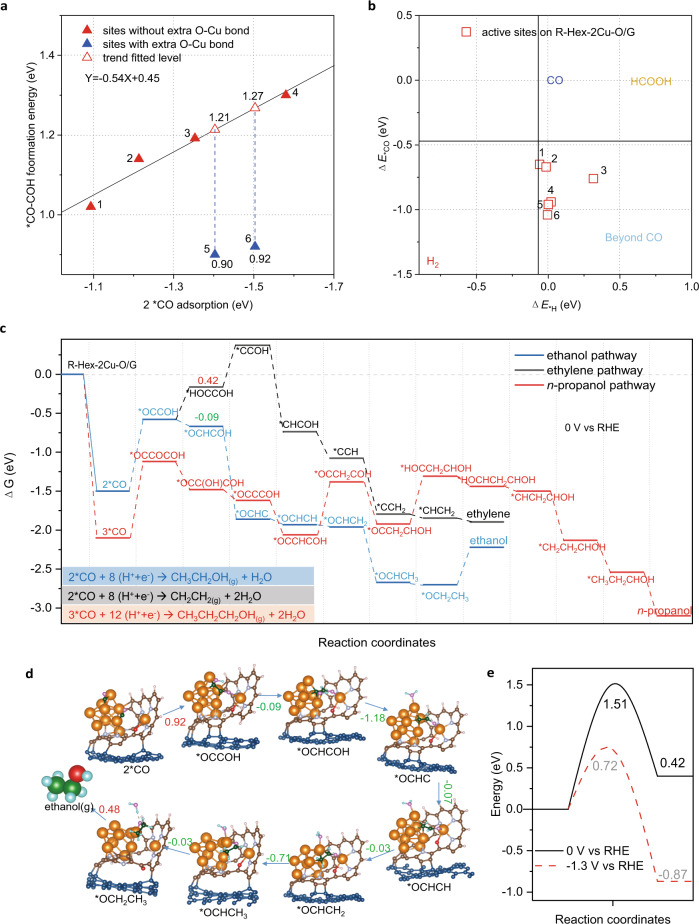
DFT calculations to unravel the formation mechanism of multi-carbon alcohols. **a** Linear scaling relations between 2*CO adsorption and *OCCOH formation free energies on R-Hex-2Cu-O/G. Atomic configuration of relevant active sites are summarized in Supplementary Fig. 35. **b** Analysis of adsorption energy for CO (ΔE_*CO_) and H (ΔE_*H_) at various sites on the R-Hex-2Cu-2O/G. Vertical and horizontal black lines represent the equilibrium states (i.e., Δ*G* = 0) for *H ↔ ½ H_2_, and *CO ↔ CO, respectively. Atomic configuration of relevant active sites are summarized in Supplementary Fig. 37. **c** Reduction pathways identified on R-Hex-2Cu-O/G at 0 V vs RHE. The reference energy level is set to be bare surface. Free energy change (Δ*G*) values for key bifurcating intermediates are annotated in eV. **d** Atomic structures of the reaction intermediates along the ethanol pathway. Δ*G* for every step at 0 V vs RHE are annotated in eV. Green and red values denote, respectively, exergonic and endergonic process. Solid blue arrows refer to the PCET steps. color codes: Cu, orange; C, brown (Hex-2Cu-O), navy (graphene) and green (reaction intermediates); O, red (Hex-2Cu-O) and purple (reaction intermediates); H, pink (Hex-2Cu-O) and blue (reaction intermediates). **e** Energy barriers of *HOCCOH formation on R-Hex-2Cu-O/G at 0 V and –1.3 V vs RHE.

We first scrutinized the key reaction intermediates for C–C coupling on R-Hex-2Cu-O/G, including two Cu-adsorbed *CO before coupling and the *OCCOH directly after coupling. For most of the active sites explored, a linear relationship exists between the two adsorption strengths (i.e., Δ*G*_2*CO_ and ΔG_*OCCOH_) as shown in Fig. 5a and Supplementary Fig. 35. However, some reaction sites significantly deviate from this linear relationship, with ΔG_*OCCOH_ decreases from the trend-fitted levels of 1.27, 1.21 eV to 0.92, 0.90 eV, respectively. These reaction sites are those within the confined-space between the Cu_9_ cluster and the adjacent single Cu center (Supplementary Fig. 35), with an extra bond formed between the adsorbed *OCCOH and adjacent Cu center (Supplementary Fig. 36a). The extra O–Cu bond boosts the *OCCOH adsorption while not affecting the 2*CO adsorption, and therefore break the scaling relation between these two carbonaceous intermediates to promote C–C coupling.

Additionally, the highly under-coordinated Cu cluster on R-Hex-2Cu-O/G can inhibit competitive hydrogen evolution reaction (HER), further contributing to its higher C_2_ selectivity. This is achieved through the significantly enhanced *CO adsorption on the under-coordinated sites. We adopted the selectivity determining method proposed by Rossmeisl et al^45^ to decide the product selectivity on R-Hex-2Cu-O/G. The results in Fig. 5b show that all active sites from the R-Hex-2Cu-O/G surface (Supplementary Fig. 37) fell in the CO_2_RR dominant zone. The calculated selectivity on the two models is in good agreement with the experimental observation of stable C_2+_ and alcohol production with suppressed H_2_ yield on Hex-2Cu-O (Fig. 2a).

After C–C coupling, the spatial-confinement effect can reserve oxygen in the hydroxyl group to increase alcohol selectivity and inhibit the competing hydrocarbon production. We acquired the complete reaction pathways toward the main products observed in experiments, including C_2_H_5_OH, C_2_H_4_ and C_3_H_7_OH. The reaction pathways are summarized in Fig. 5c; a more detailed thermodynamic profile regarding all reaction intermediates is given in Supplementary Tables 6–8. For the C_2_ pathways (blue and black), the *CO coupling and subsequent proton-coupled electron transfer (PCET) to form *OCCOH (2*CO + H^+^ + e^−^ → *OCCOH) show the maximum uphill free energy change (Δ*G*). This step is the potential-determine step (PDS) with a limiting potential (*U*_limiting_) of –0.92 V. The subsequent reduction of *OCCOH on R-Hex-2Cu-O/G proceeds in two major pathways (Fig. 5c), including: (1) C_2_H_4_ pathway (black) via the formation of intermediate *HOCCOH, and (2) C_2_H_5_OH pathway (blue) via the formation of *OCHCOH. The major difference between these two pathways is whether C or O on *OCCOH is protonated. The protonation on O to *HOCCOH is endergonic with an uphill Δ*G* = 0.42 eV, in contrast to the exergonic protonation on C to *OCHCOH (Δ*G* = –0.09 eV), which indicates that CO_2_RR can proceed preferentially toward C_2_H_5_OH formation on R-Hex-2Cu-O/G. The reason for the unfavored formation of *HOCCOH lies in the fact that the protonation on O is accompanied by the cleavage of previously formed O–Cu bond (Supplementary Fig. 38). The protonation on C to form *OCHCOH, followed by subsequent hydrogenation, can be well supported by the reaction intermediates detected by ATR-SEIRAS, especially the stretching of the C–C coupling intermediate—*OCCOH and the C_2_H_5_OH precursor—*OCH_2_CH_3_ around 1436 and 1360 cm^−1^, respectively (Fig. 4c), which in turn qualitatively validates the proposed ethanol pathway (Fig. 5d).

Our experiments observe a selectivity increase for ethylene production starting from –1.3 V on Hex-2Cu-O (Supplementary Fig. 7), which is rationalized by calculations suggesting that the accelerated reaction kinetics is led by the decreased activation barrier with the change of electrode potential. Figure 5e shows that at 0 V vs RHE the formation of *HOCCOH is kinetically unfavored on R-Hex-2Cu-O/G with an activation energy barrier of 1.51 eV (MEP shown in Supplementary Fig. 39). When applying a bias of –1.3 V, the formation of *HOCCOH becomes thermodynamically downhill (Δ*E* = –0.87 eV) with a lower activation energy barrier of 0.72 eV, leading to an acceptable reaction rate at room temperature^46^. Our calculation also confirms *CO trimerization to form *n*-propanol is facilitated by the spatial-confinement effect on R-Hex-2Cu-O/G (Supplementary Fig. 36b). The C_3_ pathway toward *n*-propanol is shown in Fig. 5c and Supplementary Fig. 40; the *U*_limiting_ is –0.98 V, similar to that for ethanol production, and therefore agrees well with the experimental observation for a notable selectivity toward *n*-propanol (Fig. 2a). Nonetheless, it is worth to note that given the complex phase space demonstrated by R-Hex-2Cu-2O/G that would require as-of-yet unrealized simulation capability to conclusively depict, the mechanism proposed here is pending for further clarification to understand the causal factors from electronic structure and ultimately the contributions of structural evolution to the observed kinetics.

In short, our calculations show the highly under-coordinated Cu cluster in the hybrid inorganic/organic structure suppresses HER by enhancing *CO adsorption, and thereby promotes C–C coupling to form *OCCOH, which is the PDS. Furthermore, the confined space between the Cu cluster and adjacent single Cu center affords an extra O–Cu bond to stabilize the *OCCOH intermediate by breaking the linear scaling relation with *CO. Next, CO_2_RR proceeds preferentially toward C_2_H_5_OH formation through the exergonic protonation on C to from *OCHCOH, whereas the protonation on O to form *HOCCOH is disfavored owing to the extra energy needed to cleave the O–Cu bond. Lastly, the electrochemical CO_2_RR of Hex-2Cu-O was further tested in a flow-cell using the aqueous 1 M KOH electrolyte. Similar trend of ethanol, propanol, and ethylene was observed with a noticeable amount of acetic acid (Supplementary Fig. 41a). At the current density of 250 mA cm^−2^, the total FE of oxygenates (including ethanol, propanol, and acetic acid) sums up to 38.6% and that of C_2+_ products reaches 55.4%. Chronoamperometric *i–t* test at −0.66 V (Supplementary Fig. 41b) shows the catalyst is able to maintain a stable current density of ∼280 mA cm^−2^ for up to 5 h, with the FE of total oxygenates kept at ∼36% throughout the testing period. Moreover, comprehensive characterizations using XPS (Supplementary Fig. 42), MALDI-TOF MS (Supplementary Fig. 43) and HR-TEM (Supplementary Fig. 44) all point to the coexistence of Cu nanoclusters and partially reduced Hex-2Cu-O complexes in the post-electrolytic catalyst, similar to the scenario in H-cell tests.

## Discussion

In this study, bicentric complexes of Hex-2Cu-O, Hex-2Cu-2O and Oct-2Cu with different molecular configuration and coordination geometry are employed as CO_2_RR catalysts to unveil the underlying structure-property-performance relationship. Owing to the shared oxygen atom and thus caused intramolecular tension, Hex-2Cu-O manifests a low electrochemical stability with highly redox-active Cu centers, which can be restructured during CO_2_RR to form inorganic/organic hybrids comprising under-coordinated Cu clusters and partially reduced hexaphyrin complexes. This hybrid structure in turn creates a synergy to drastically promote multi-carbon products during CO_2_RR, especially with high FE of oxygenates that has been rarely seen in the literature. By contrast, the planar Hex-2Cu-2O with independent Cu–N_3_O motifs exhibits a much better electrochemical stability, but only mediocre CO_2_RR performance producing majorly H_2_ and HCOOH. Due to ligand fragmentation, Oct-2Cu mainly exhibits the CO_2_RR performance of Cu clusters. DFT calculations further draw a vivid picture on the collaboration between the under-coordinated Cu cluster and vicinal Cu center to synergistically promote C–C coupling and oxygenation by providing a confined space to afford extra O–Cu bonding. Consequently, this study sheds new light on tuning the molecular configuration and coordination geometry to impart structure-dependent electrochemical property, which in turn steers the CO_2_RR pathway. What’s more, it might offer a general tactic to target alcohol synthesis in CO_2_RR by constructing organic/inorganic Cu hybrids.

## Methods

### Chemicals

Cloromethane (C_2_Cl_2_H_2_, ≥99.7%) and Hexyl hydride (C_6_H_14_, ≥99.7%) were purchased from Sinopharm Chemical Reagent Co., Ltd. Sodium acetate trihydrate (C_2_H_3_O_2_Na·3H_2_O, ≥99.5%), cupric acetate anhydrous (Cu(C_2_H_3_O_2_)_2_) and pyrrole (C_4_H_5_N, ≥99.5%) were obtained from Shanghai Aladdin Blochemical Technology Co., Ltd. Pentafluorobenzaldehyde (C_7_HF_5_O, ≥98%), Cuprous chloride (CuCl·2H_2_O, ≥97%) and 2,3-Dichloro-5,6-dicyano-1,4-benzoquinone (C_8_Cl_2_N_2_O_2_, DDQ) were provided by Shanghai Macklin Biochemical Co., Ltd. Boron trifluoride diethyl etherate (C_4_H_10_BF_3_O, ≥98%) was provided by Shanghai Yien Chemical Technology Co., Ltd. Pyridine (C_5_H_5_N, ≥99.5%) was provided by Chinasun Specialty Products Co., Ltd. Copper(II) Chloride Dihydrate (Cl_2_CuH_4_O_2_, ≥99.7%) was provided by Shanghai Titanchem Co., Ltd. Potassium bicarbonate (KHCO_3_, ≥99.5%) was purchased from J&K Scientific Co., Ltd. Ethanol (C_2_H_6_O, ≥99.7%) was provided by Shanghai Lingfeng Chemical Reagent Co., LTD. The CO_2_ gas (99.995%) was supplied by Suzhou Jinhong Gas Co., Ltd. All materials were used as received without further purification. Deionized (DI) water was purified with a Sartorius arium mini ultrapure water system.

### Synthesis of hexaphyrin and octaphyrin

Hexaphyrins(1.1.1.1.1.1) were prepared following the reported method by Osuka et. al with a slight modification. Dichloromethane (DCM) (200 mL) was added to a 500 mL single-neck round-bottom flask filled with nitrogen. Steamed pyrrole (1.35 g, 0.02 mol, 1.39 mL) and pentafluorophenaldehyde (3.92 g, 0.02 mol, 2.57 mL) were then added and stirred vigorously at room temperature. After 5 minutes, boron trifluoride diethyl etherate (1.13 g, 3.96 mmol, 1 mL) was added and stirred for 2 h under dark conditions. Excess DDQ (11.4 g, 0.05 mol) was added to the reaction solution and continued to stir overnight. Afterwards, the dark red reaction mixture was directly pumped through filter membrane to remove excessive porphyrin. The filtrate was separated by silica gel column chromatography. By using 30 % DCM in hexane, blue fraction corresponding to octaphyrin (0.12 g, 2.3%) was obtained. The filter residue was then washed with water to remove the residual DDQ, attaining the crude product. Finally, hexaphyrin (0.25 g, 4.7%) in the filtrate was obtained by washing the crude product with methylene chloride.

### Synthesis of hexaphyrin–copper complex

The hexaphyrin-copper complex, Hex-2Cu-O, was prepared as following: In a 100 mL round-bottom flask, hexaphyrin (0.086 g, 0.06 mmol) was added and dissolved in chloroform (40 mL). The reaction was initiated by the addition of Cu(OAc)_2_ (0.12 g, 0.60 mmol) and NaOAc·3H_2_O (0.082 g, 0.60 mmol) in methanol (10 mL). The mixture was refluxed overnight and then the solvent was evaporated under reduced pressure. Subsequently, the obtained solid was dissolved in chloroform (5 mL) and purified by silica-gel chromatography with 30% n-hexane in chloroform as the eluent, yielding the target product in the first bluish-gray band. The collected fractions were concentrated and recrystallized in dichloromethane/n-hexane to obtain black crystals (0.086 g, 90%).

### Synthesis of N-confused hexaphyrin–copper complex

The N-confused hexaphyrin-copper complex, Hex-2Cu-2O, was prepared as follows: Hexaphyrin (200 mg, 0.14 mmol) and CuCl·2H_2_O (1 g, 5.87 mmol) in pyridine (50 mL) were stirred under oxygen flow for 3 h at room temperature. The reaction mixture was then diluted with dichloromethane, and the organic layer was washed with saturated copper chloride aqueous solution three times. The obtained crude product was washed by methylene chloride to remove the pyridine complex. After removal of solvent, the crude product was purified by silica gel column chromatography with CH_2_Cl_2_/hexane (3:7) as the eluent. The major blue-green fraction was collected. Recrystallization from a mixture of CH_2_Cl_2_/hexane gave the reddish solids of Hex-2Cu-2O (50 mg, 22%).

### Synthesis of octaphyrin–copper complex

The bicentric copper-octaphyrin complex, Oct-2Cu, was prepared by a reflux reaction at room temperature. 100 mg octaphyrin was dissolved in 50 mL of dichloromethane, in which 100 mg copper acetate was added. The reflux reaction lasted for 72 h, and then the reaction solution was concentrated and purified to obtain 60 mg Oct-2Cu.

### Characterizations

UV–Vis absorption measurements were carried out with a Techcomp UV2300-II UV–Vis spectrophotometer. Mass spectra were recorded using a Bruker Ultraflextreme MALDI-TOF instrument. The surface composition and valence states were analyzed with XPS, using an Escalab 250Xi X-ray photoelectron spectrometer (Thermo Fisher) with Al Ka (1486.6 eV) X-rays as the excitation source, and the binding energy of the C 1 *s* peak at 284.8 eV was taken as an internal reference. The pass energy was 30 eV and the photoemission angle was 45°. The energy linearity detection was calibrated with Au 4 *f* (83.96 eV), Ag 3*d*5 (368.21 eV) and Cu 2*p* (932.62 eV), respectively. 0.4 mg of catalyst in the powder form was loaded onto 0.25 × 0.5 cm^2^ of carbon paper for XPS testing. The electron paramagnetic resonance (EPR) experiments were carried out on a JES-X320 spectrometer (Japan Electron Optics Laboratory). The catalyst microstructure was characterized by an FEI Talos F200X field-emission TEM equipped with EDX analyzer and operated at 200 KV. The atomic arrangement of post-electrolytic Hex-2Cu-O was examined by Spherical-aberration-corrected TEM (Cs-TEM, FEI Titan Themis Cubed G2 300). The operando XAS spectra at the Cu K-edge were recorded at the BL11B beamline of Shanghai Synchrotron Radiation Facility (SSRF) by following the same procedure detailed in our previous study^34^, with the electron storage ring operated at 3.5 GeV. The beam current of the storage ring was set to 220 mA in a top-up mode.

### Electrochemical measurements

The CO_2_ electroreduction experiments were carried out in both H-cell and flow-cell controlled by an electrochemical workstation (CHI660E). The two compartments of the H-cell, each containing 30 mL of 0.1 M KHCO_3_, were separated by an ion-exchange membrane (Nafion perfluorinated membrane). A carbon rod was used as the counter electrode, while an Ag/AgCl electrode was used as the reference electrode. The reference electrode was calibrated before each test. Slurries of catalyst ink were prepared through the sonication of 4 mg sample powder, 1 mg KB, and 50 µL (5%) Nafion solution in 1 mL ethanol. Then, 10 µL of the catalyst ink was drop-casted onto a glassy carbon electrode with a surface area of 0.197 cm^2^. The as-obtained working electrode was then fully dried at 50 °C for subsequent testing. Prior to the electrochemical measurement CO_2_ was purged into the cathode chamber for at least 30 min to saturate the electrolyte. During measurement, CO_2_ at a constant flow rate of 20 cm^3^ min^−1^, controlled by a digital mass flow controller, was kept bubbling into the electrolyte. The activation of the working electrode was carried out by conducting cyclic voltammetry in the potential range of −0.4 to −1.4 V at a rate of 10 mV s^−1^ for 20 min.

The flow cell is composed of three chambers, including the gas chamber, catholyte chamber and anolyte chamber. While the gas and catholyte chambers are separated by the gas diffusion electrode (YLS−30T), the catholyte and anolyte chambers are separated by the anion exchange membrane (Fumasep FAB-PK-130). The Ag/AgCl reference electrode was inserted into the catholyte chamber and a nickel foam of 0.8 mm in thickness was used as the counter electrode for water oxidation. The catalyst slurry was sprayed onto the side of the gas diffusion electrode facing the catholyte with an areal loading of 0.8 mg cm^−2^. Silicone gasket with a 1 × 1 cm^2^ window was used to seal each chamber to avoid gas and liquid leakage. High-purity CO_2_ was supplied to the gas chamber at a constant flow rate of 30 cm^3^ min^−1^ controlled a digital mass flow controller (Horiba). 1 M KOH was used as both the catholyte and anolyte circulated at a constant flow rate of 20 ml min^−1^ via a two-channel peristaltic pump. All potentials were converted to RHE, according to the formula was *E* (vs RHE) = *E* (vs Ag/AgCl) + 0.059 pH + 0.198. Unless otherwise noted specifically, all electrochemical data were iR-uncompensated.

The gas products were quantified by an Agilent 7890B gas chromatography equipped with both the flame ionization detector (FID) and the thermal conductivity detector (TCD). The liquid products were quantified through ^1^H NMR (600 MHz Agilent DD2 NMR spectrometer) using dimethyl sulfoxide as the internal standard with water inhibition. The NMR samples were prepared by adding 50 μL DMSO solution and 100 μL D_2_O into 400 μL of the electrolyte solution. All product concentrations were quantified by integrating the GC and NMR peak area.

### Operando ATR-SEIRAS measurements

Generally, a layer of 100 nm Au film was deposited onto the reflecting plane of a Si prism by vacuum evaporation using a thermal evaporator (PuDi vacuum PD-400). Before the Au deposition, the Si prism was polished with 0.05 μm Al_2_O_3_ suspension and cleaned by sonication sequentially in the baths of acetone and deionized water. The working electrode was made by airbrushing the catalyst ink onto the above prepared Au film. The ATR-SEIRAS measurements was conducted in a two-compartment spectroelectrochemical cell comprising three electrodes including the working electrode, a platinum-wire as the counter electrode and a standard Ag/AgCl electrode as the reference. All the ATR-SEIRAS spectra were acquired using a Fourier Transform Infrared Spectrophotometer (FT-IR, Nicolet iS50, Thermo Fischer scientific.) equipped with a mercury cadmium telluride (MCT) detector. All electrochemical tests were tested in 0.5 M KHCO_3_ aqueous solution with a constant CO_2_ flow, and controlled by a CHI electrochemical workstation (CHI760E). In a typical test, the working electrode was subjected to an initial activation by running CV cycles between −0.1 and −1.4 V vs RHE at a scan rate of 0.05 V S^−1^ until the system was stabilized. The spectrum under open circuit voltage was then collected as the background. The cathode potential was then swept from −0.5 V to −1.3 V vs RHE, with each potential lasting 2 minutes for spectra acquisition. All measurements were performed at a spectral resolution of 4 cm^−1^ and presented in transmission units after subtracting the background.

### Computational methods

All DFT calculations were performed with the Vienna Ab Initio Simulation Package (VASP) code^47^. The Perdew–Burke–Ernzerhof (PBE) was employed for electron exchange-correlation^48^. Projector Augmented Wave (PAW) potentials were used to describe the ionic cores^49^. The atomic relaxations were carried out with the quasi-Newton minimization scheme until the maximum force on any atom was below 0.03 eV/Å. The geometry optimizations were performed with a plane-wave cutoff of 400 eV. Irreducible 2 × 2 × 1 Monkhorst Pack k-point grid was used^50^, with the centre shifted to the gamma point. A Gaussian smearing was employed with a smearing width of 0.05 eV. The vertical separation between periodically repeated images was set to be at least 15 Å in all cases, to ensure no interaction between images.

The implicit solvent effect was considered by using VASPsol^51^. Within this model, the solvent dielectric constant was set to be 78.4 F/m, with width of dielectric cavity 0.6 Å, cutoff charge density 2.5 × 10^–3^ C/m^3^, and the effective surface tension 5.25 × 10^–4^ N/m.

The DFT-calculated electronic energies (*E*) are converted into free energies in the following way:

1 $$\begin{array}{c}G=E+{ZPE}-{TS}\end{array}$$

where ZPE is the zero-point energy correction and *TS* is the entropy correction at room temperature (300 K). The free energies of relevant gas molecules are given in Supplementary Supplementary Table 9.

Limiting potential (*U*_limiting_) was applied to describe the lowest potential requirement to eliminate the free energy difference of the potential-determining step (PDS), as described as:

2 $$\begin{array}{c}{U}_{{{{{{{\rm{limiting}}}}}}}}=-\frac{\varDelta {G}_{{\max }}}{e}\end{array}$$

Activation energy barrier (*E*_a_) is essential to determine the product selectivity and dominant reaction pathway. This was computed using the nudged elastic band (NEB) approach^52^. The total energy and force thresholds for geometry optimizations were 1 × 10^−5^ eV and 0.05 eV/Å, respectively. The minimum energy pathway (MEP) was examined using six images during the transition state search. Each transition state was confirmed to have a single imaginary vibrational frequency along the reaction coordinate, as shown in Supplementary Supplementary Table 10. DFT-calculated activation energy barrier *E*_a_(*U*_0_) can be extrapolated to potential *U* by the following equation^53^:

3 $$\begin{array}{c}{E}_{a}\left(U\right)={E}_{a}\left({U}_{0}\right)+{{{{{\rm{e}}}}}}{\beta }^{{\prime} }\left(U-{U}_{0}\right)\end{array}$$

where *U*_0_ is the equilibrium potential for the relevant elementary reduction reaction step, and β’ is the reaction symmetry factor, which was approximated to be 0.49 for the models in this work^54^.

## Supplementary information

Supporting Information

## Data Availability

The data that support the findings of this study are available from the authors upon request.
