# Supplementary material for: Electrocatalytic CO2 reduction to alcohols by modulating the molecular geometry and Cu coordination in bicentric copper complexes
Source: Nat Commun. 2022 Aug 31;13:5122. doi: 10.1038/s41467-022-32740-z (PMC9433389; doi:10.1038/s41467-022-32740-z)
Supplement: Supplementary file 1 — Supporting Information [file 41467_2022_32740_MOESM1_ESM.pdf]

Supporting Information

Electrocatalytic CO_2 Reduction to Alcohols by Modulating the Molecular Geometry and Cu Coordination in Bicentric Copper Complexes

Baiyu Yang[1,2,5], Ling Chen[3,5], Songlin Xue[1], Hao Sun[1,2], Kun Feng[4], Yufeng Chen[4], Xiang Zhang[1,2], Long Xiao[1,2], Yongze Qin[1,2], Jun Zhong[4], Zhao Deng[1,2]*, Yan Jiao[3]*, and Yang Peng[1,2]*

[1]Soochow Institute for Energy and Materials Innovations, College of Energy, Key Laboratory of Advanced Carbon Materials and Wearable Energy Technologies of Jiangsu Province, Soochow University, Suzhou 215006, P. R. China.

[2]Jiangsu Key Laboratory of Advanced Negative Carbon Technologies, Soochow Municipal Laboratory for Low Carbon Technologies and Industries, Soochow University, Suzhou, 215123, Jiangsu, P. R. China.

[3]School of Chemical Engineering and Advanced Materials, The University of Adelaide, Adelaide SA 5005, Australia.

[4]Institute of Functional Nano & Soft Materials (FUNSOM), Jiangsu Key Laboratory for Carbon-Based Functional Materials & Devices, Soochow University, Suzhou 215123, China.

[5]These authors contributed equally: Baiyu Yang, Ling Chen. Correspondence and requests for materials should be addressed to Z.D. (email: zdeng@suda.edu.cn) or to Y.J. (yan.jiao@adelaide.edu.au) or to Y.P. (email: ypeng@suda.edu.cn)

Table of Contents

1. Supplementary Figures and Tables

2. References

Methods

Faradaic Efficiency of Gas Products.

$$FE_a = \frac{\frac{v \times C_a}{A \times v_m} \times z_a \times F}{j_{total}} \times 100\%$$

FE_a: Faradaic Efficiency of the product a

v: CO_2 gas flow rate (L s^{-1})

C_a: Volumefraction of the product a detected by GC

A: Geometric area of the electrode (cm^{-2})

V_m: molar Volume (22.4 L mol^{-1})

Z_a: electrons transferred for reduction to product a

F: Faradaic Constant (C mol^{-1})

j_{total}: Total current density during CO_2 bulk electrolysis (A cm^{-2})

Partial current density.

$$j_a = FE_a \times j_{total}$$

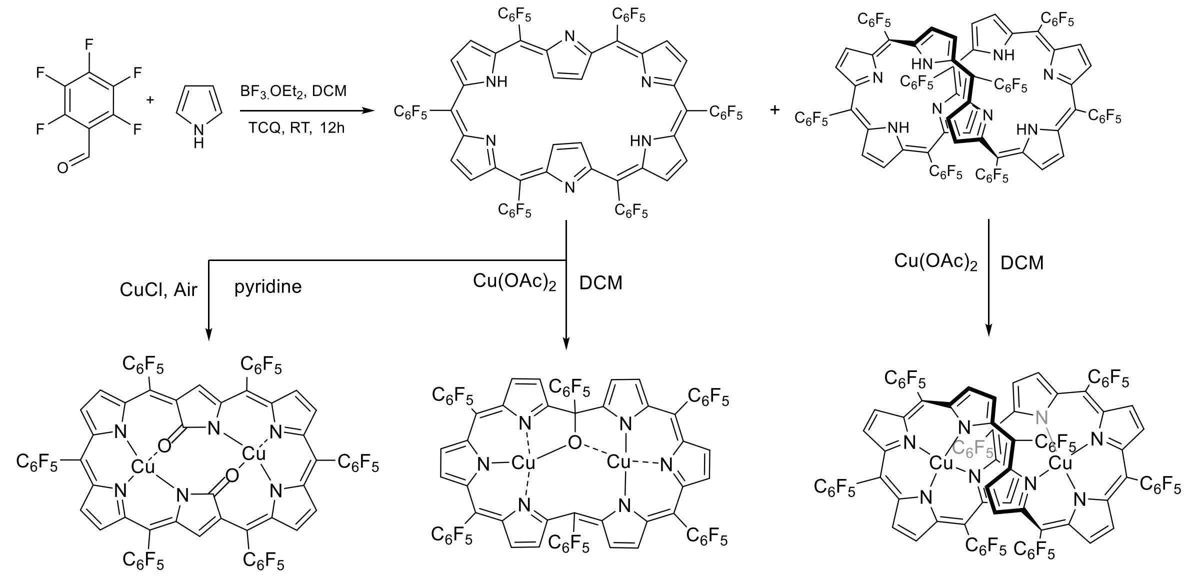

Supplementary Fig. 1 Synthesis of the bicentric copper complexes Hex-2Cu-O, Hex-2Cu-2O and Oct-2Cu.

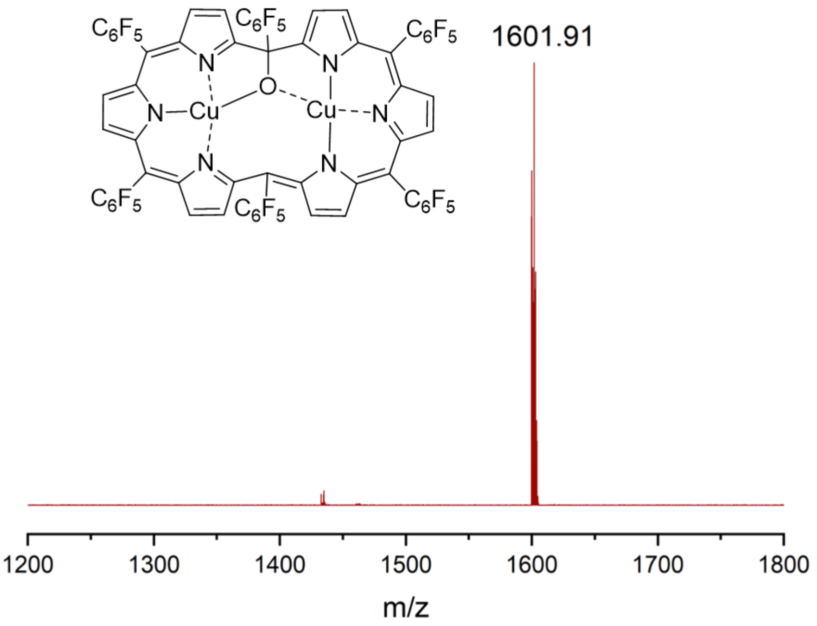

Supplementary Fig. 2 MALDI-TOF MS spectrum of Hex-2Cu-O.

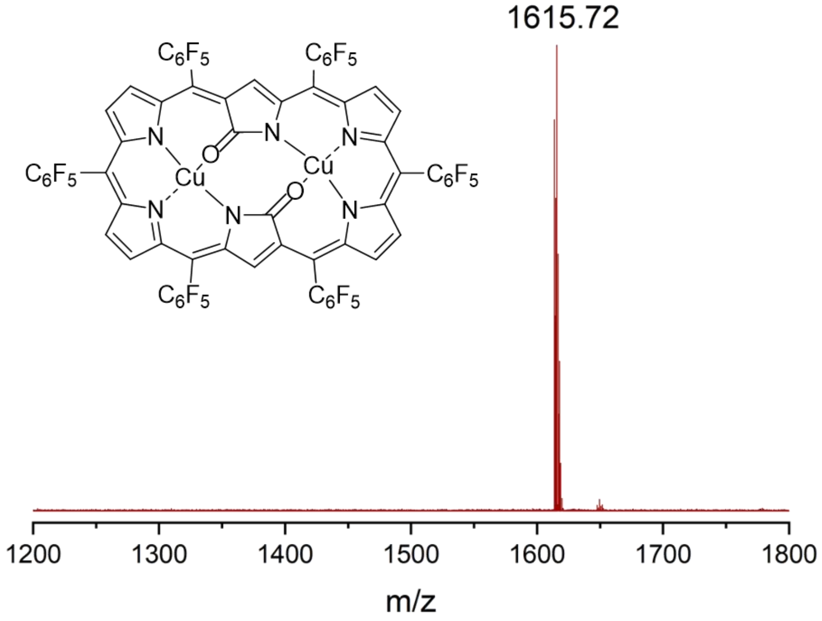

Supplementary Fig. 3 MALDI-TOF MS spectrum of Hex-2Cu-2O.

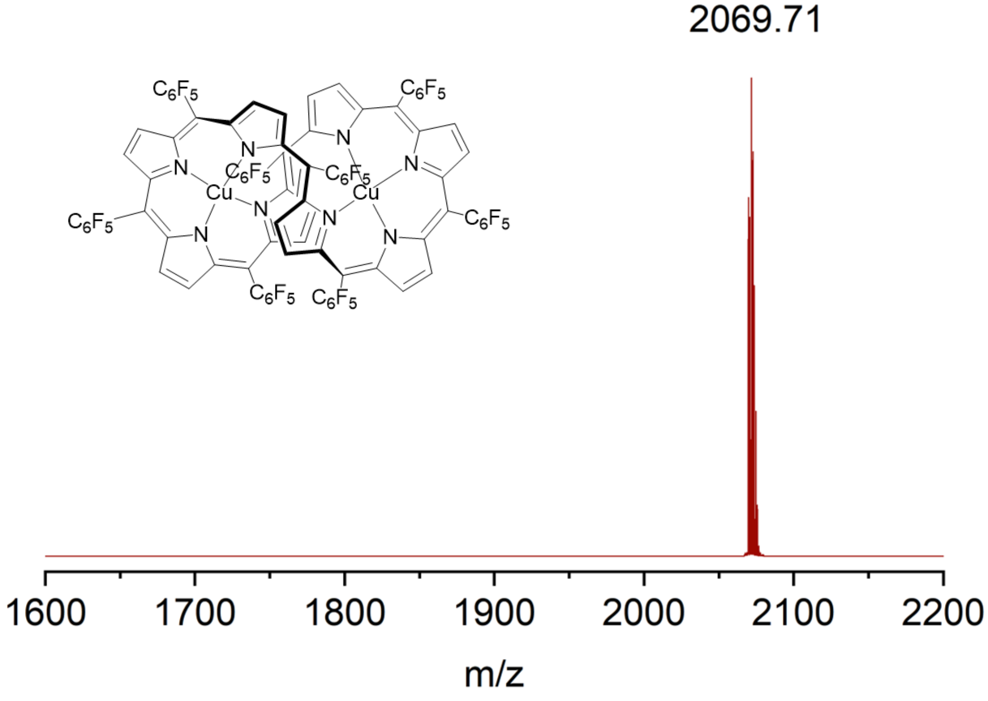

Supplementary Fig. 4 MALDI-TOF MS spectrum of Oct-2Cu.

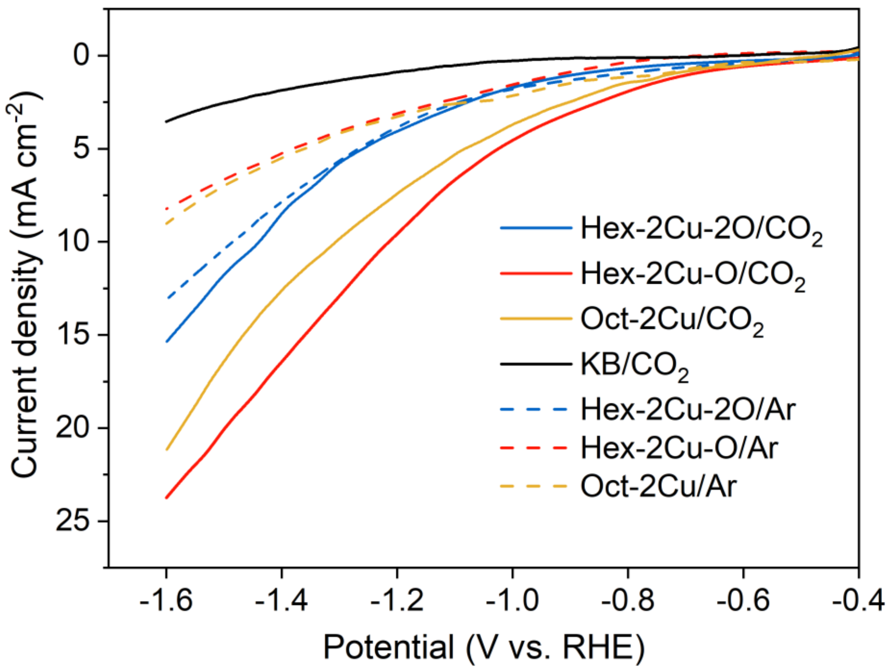

Supplementary Fig. 5 Polarization curves recorded under CO_2 and Ar. LSVs of Hex-2Cu-O, Hex-2Cu-2O and Oct-2Cu in CO_2 and Ar-saturated 0.1 M $KHCO_3$ within the potential range from -0.4 to -1.6 V vs. RHE.

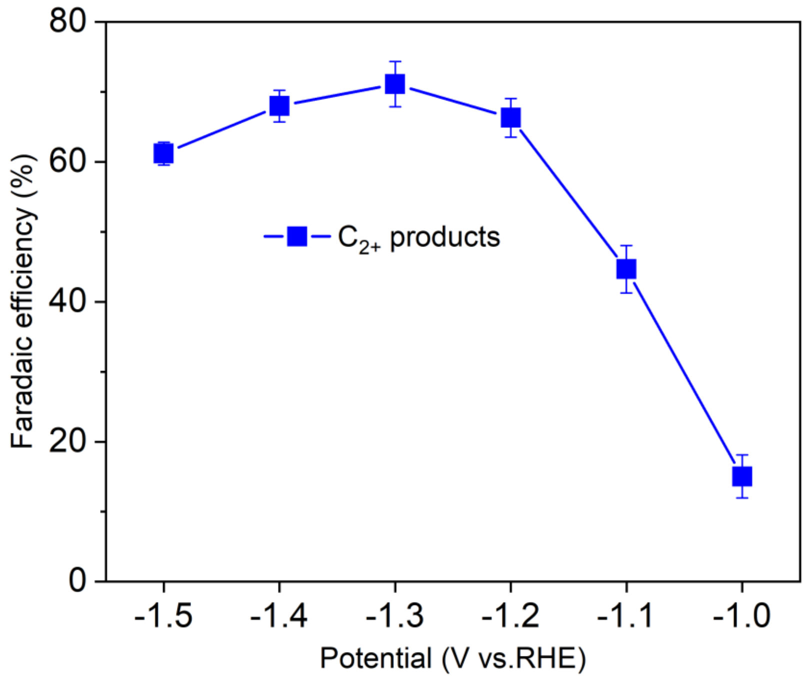

Supplementary Fig.6 Faradaic efficiencies of C_{2+} products catalyzed by Hex-2Cu-O at various potentials. Error bars represent the standard deviation of three independent measurements.

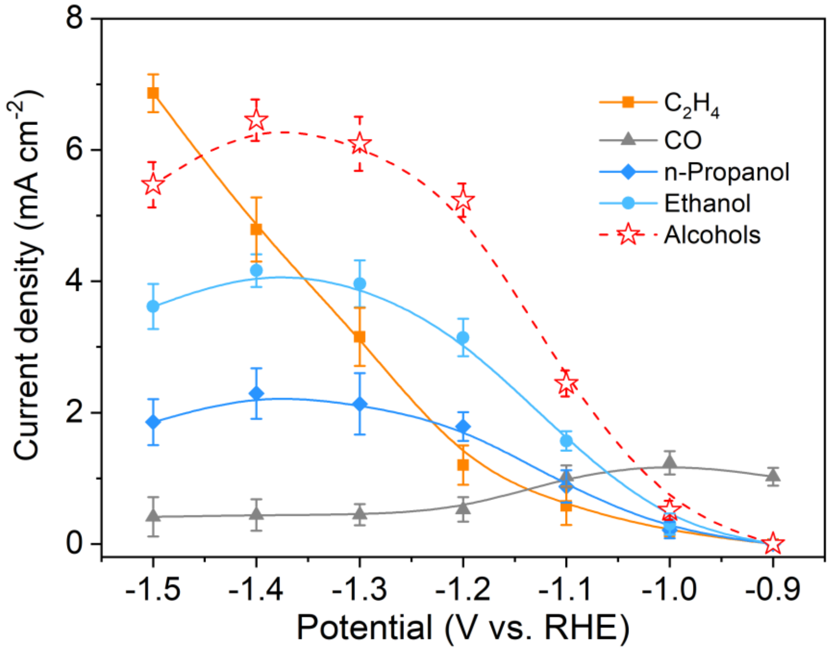

Supplementary Fig.7 Partial current densities of various CO_2RR products catalyzed by Hex-2Cu-O. Error bars represent the standard deviation of three independent measurements.

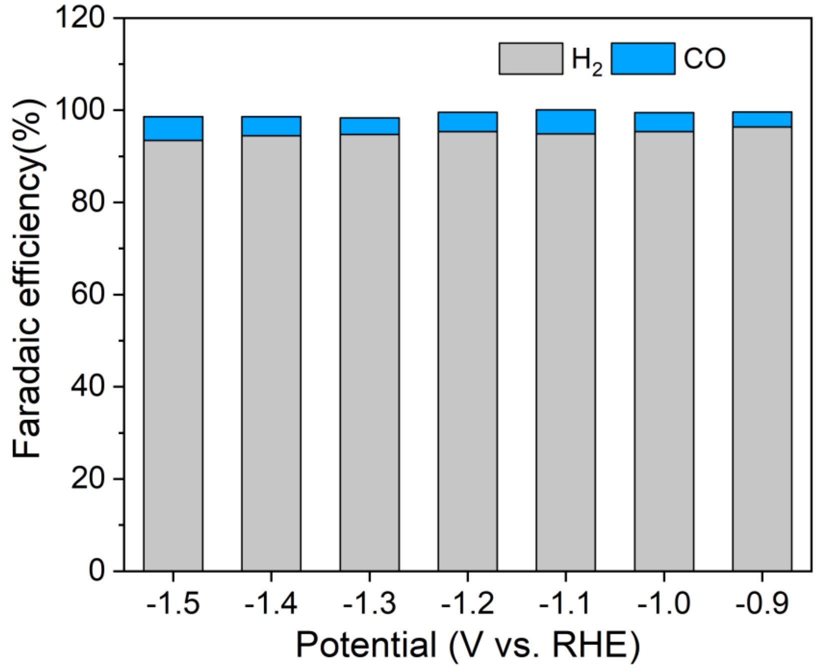

Supplementary Fig.8 Electrochemical performance of KB. Faradaic efficiency of reduction products catalyzed by KB alone at various potentials in CO_2-saturated 0.1 M $KHCO_3$ electrolyte.

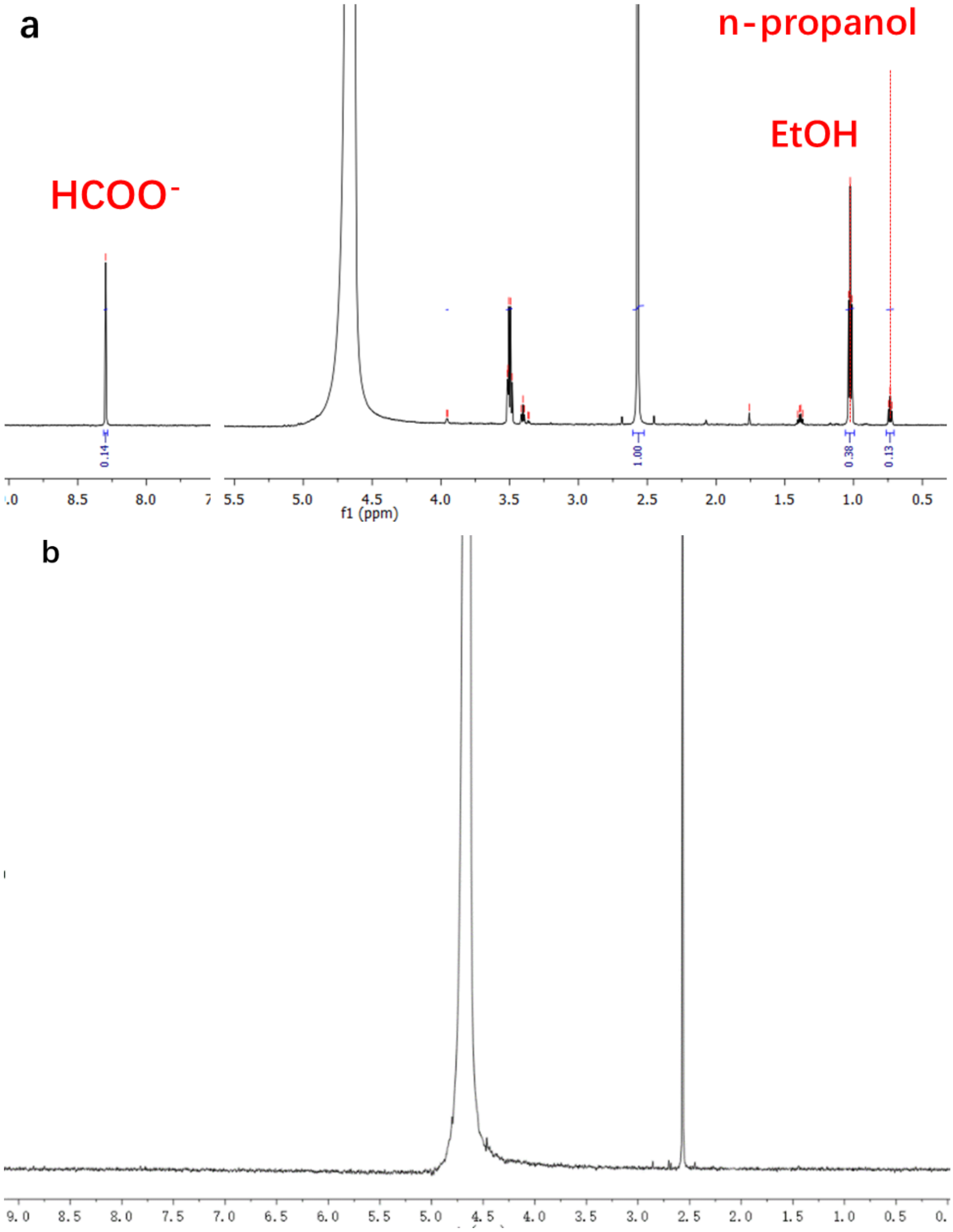

Supplementary Fig. 9. ^{1}H-NMR spectra of the electrolyte. Liquid products catalyzed by Hex-2Cu-O after 20 hours of electrolysis at −1.2 V vs RHE in (a) CO_2- and (b) Ar-purged electrolytes with DMSO serving as the internal standard.

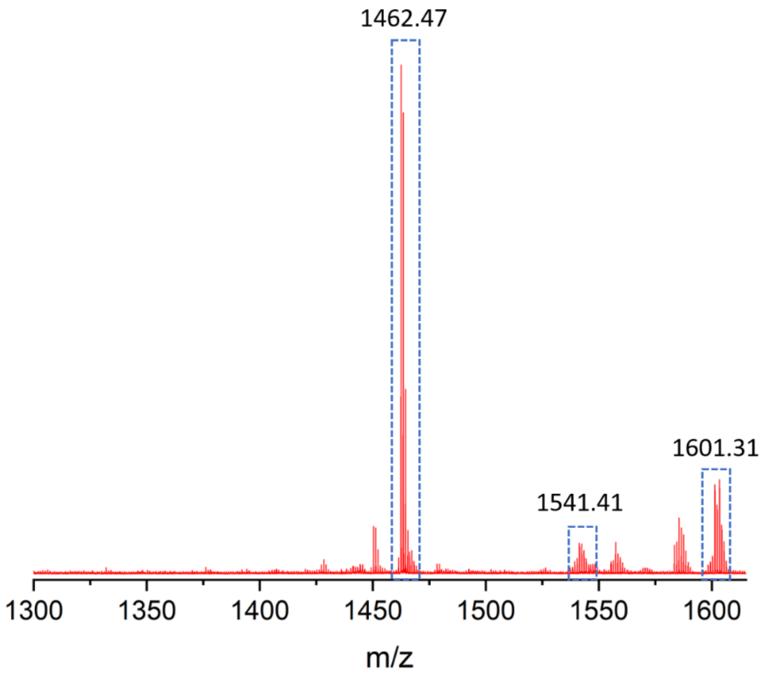

Supplementary Fig. 10 MALDI-TOF MS spectrum of the post-electrolytic Hex-2Cu-O after 10 h of CO_2RR at -1.2 V vs RHE.

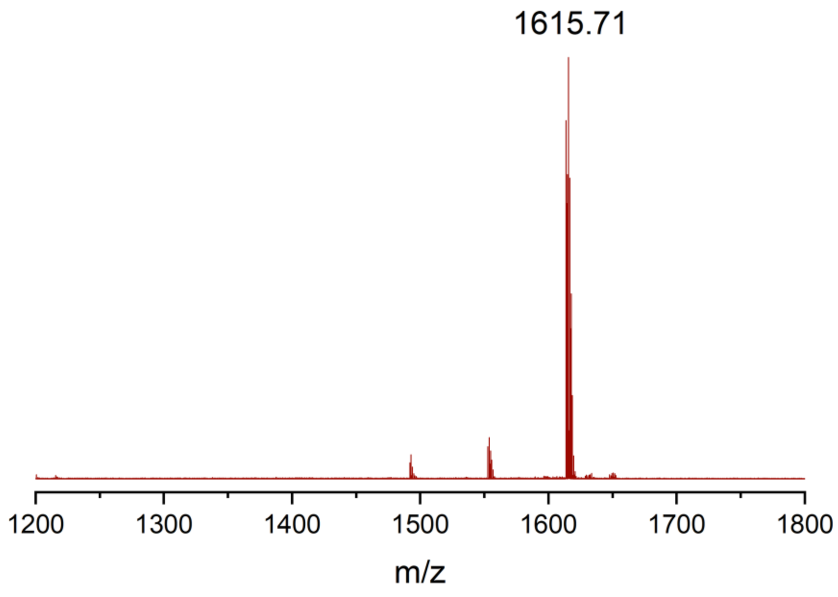

Supplementary Fig. 11 MALDI-TOF MS spectrum of the post-electrolytic Hex-2Cu-2O after 10 h of CO_2RR at -1.2 V vs RHE.

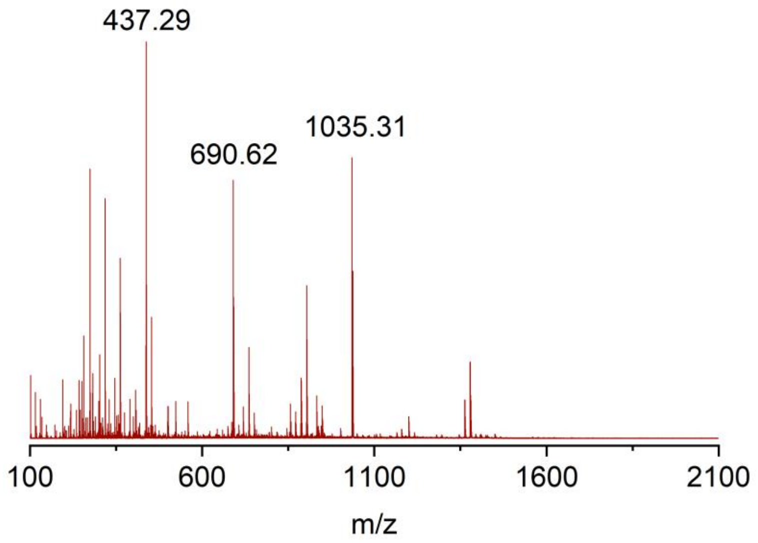

Supplementary Fig. 12 MALDI-TOF MS spectrum of the post-electrolytic Oct-2Cu after 10 h of CO_2RR at -1.2 V vs RHE.

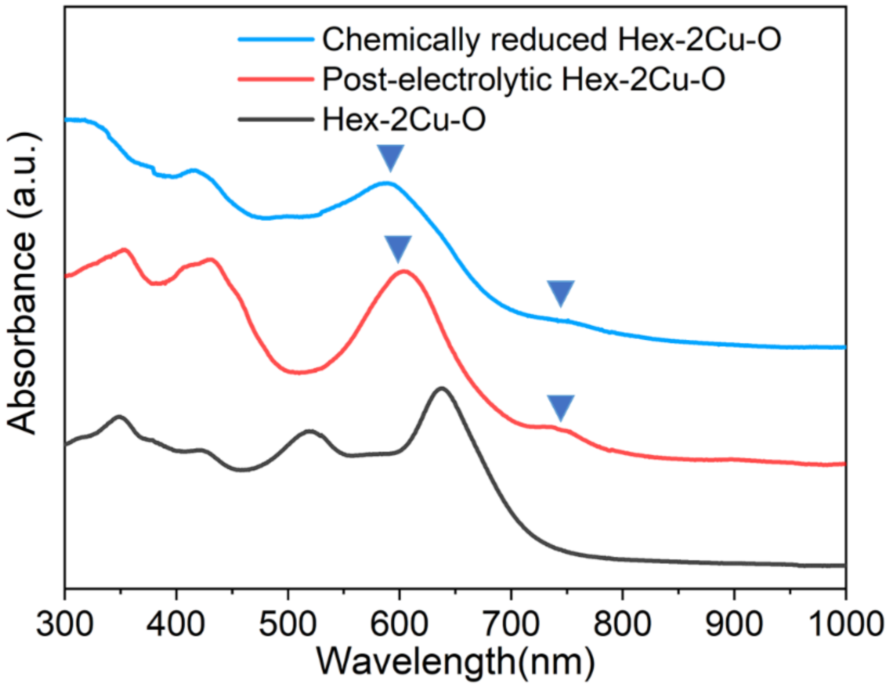

Supplementary Fig. 13 UV-Vis spectra of the pristine, post-electrolytic and chemically reduced Hex-2Cu-O.

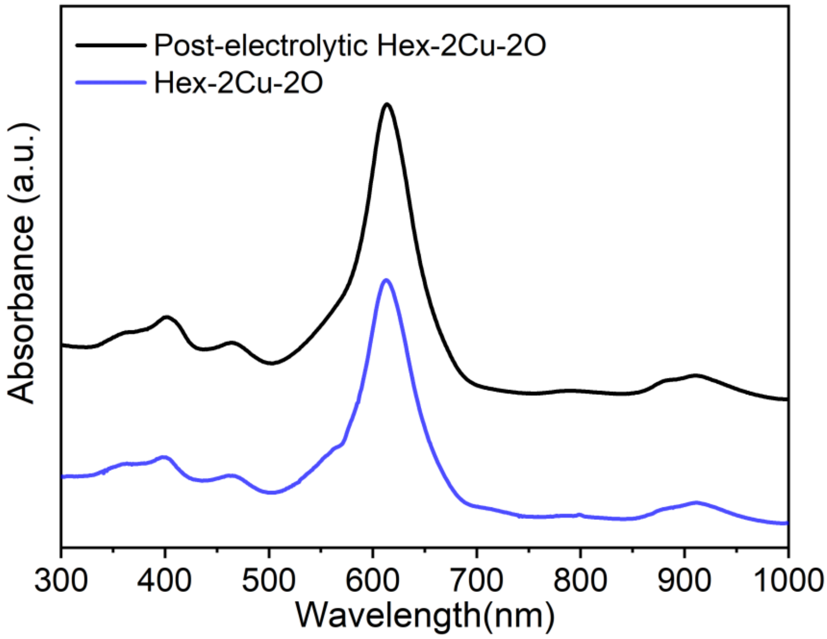

Supplementary Fig. 14 UV-Vis spectra of the pristine and post-electrolytic Hex-2Cu-2O.

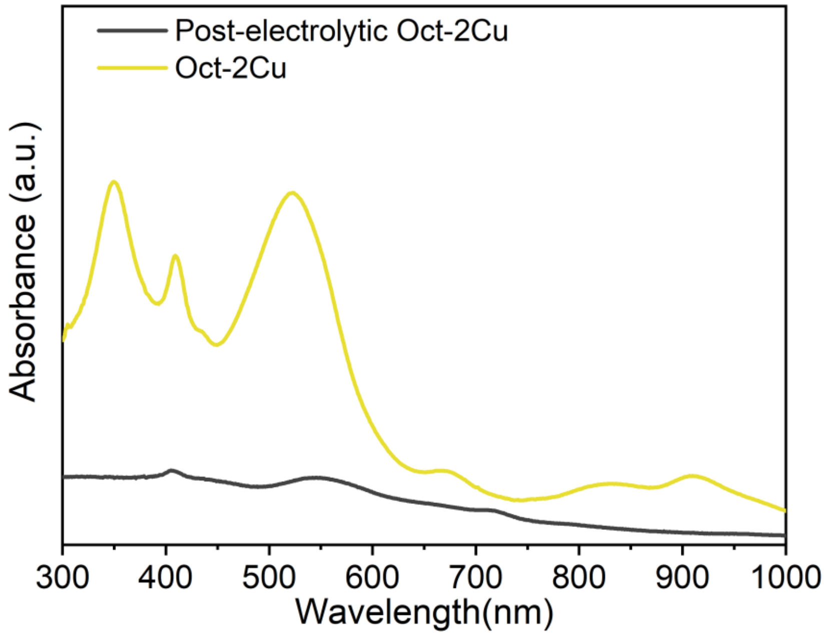

Supplementary Fig. 15 UV-Vis spectra of the pristine and post-electrolytic Oct-2Cu.

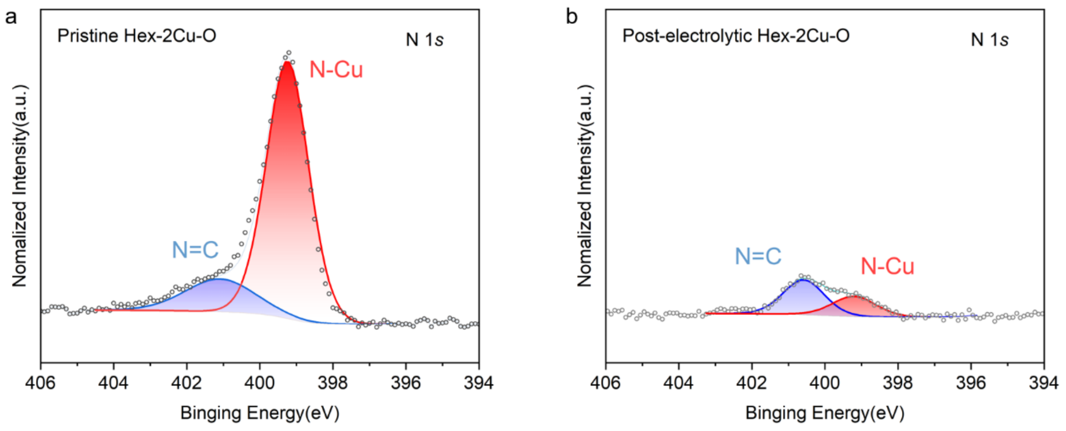

Supplementary Fig. 16 XPS N 1*s* spectra of Hex-2Cu-O. (a) Pristine and (b) post-electrolytic Hex-2Cu-O.

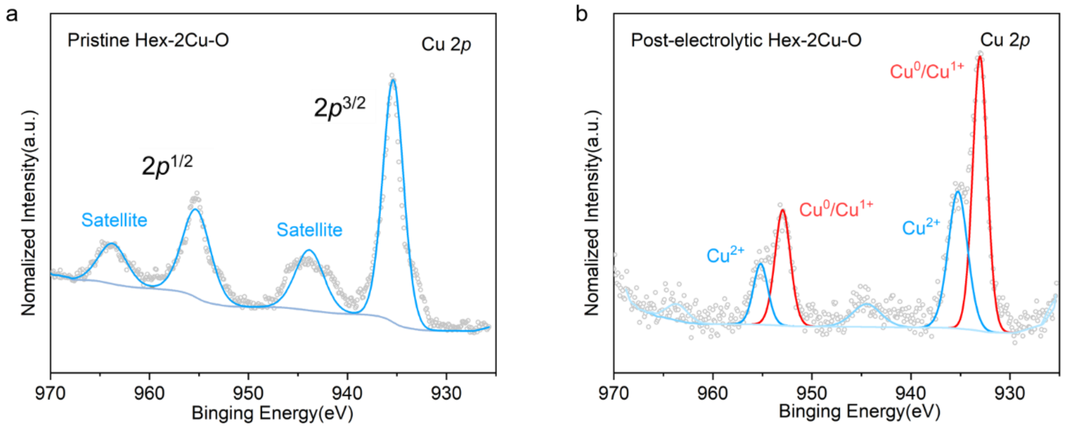

Supplementary Fig. 17 XPS Cu 2*p* spectra of Hex-2Cu-O. (a) Pristine and (b) post-electrolytic Hex-2Cu-O.

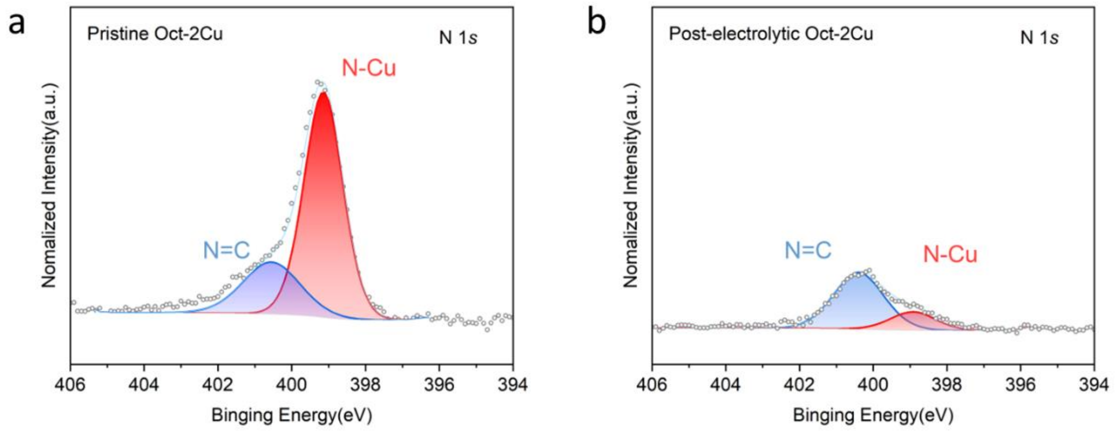

Supplementary Fig. 18 XPS N 1*s* spectra of Oct-2Cu. (a) Pristine and (b) post-electrolytic Oct-2Cu.

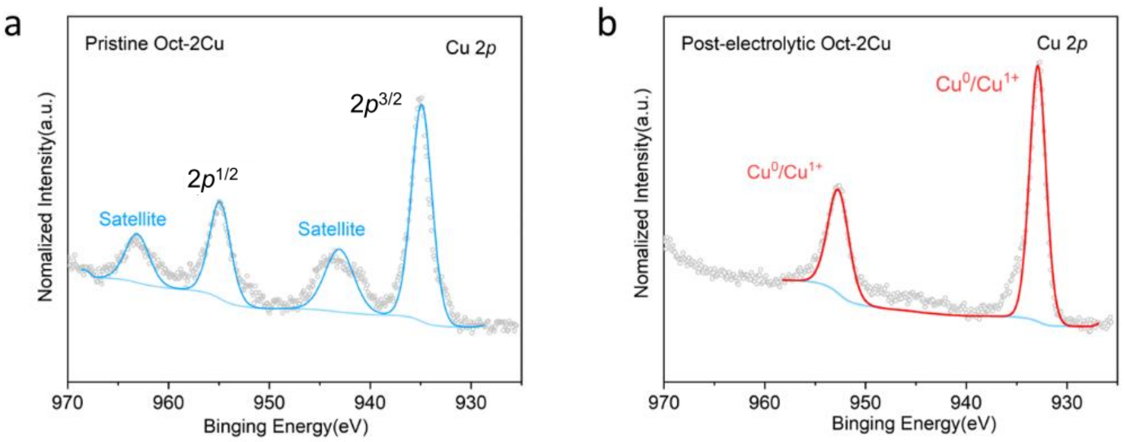

Supplementary Fig. 19 XPS Cu 2*p* spectra of Oct-2Cu. (a) Pristine and (b) post-electrolytic Oct-2Cu.

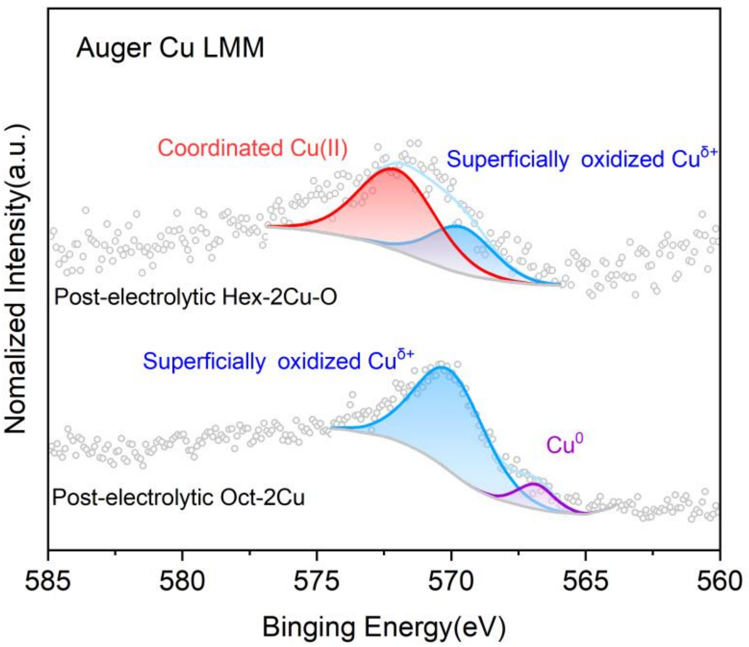

Supplementary Fig. 20 Auger Cu LMM spectra of post-electrolytic Hex-2Cu-O (top) and Oct-2Cu (bottom).

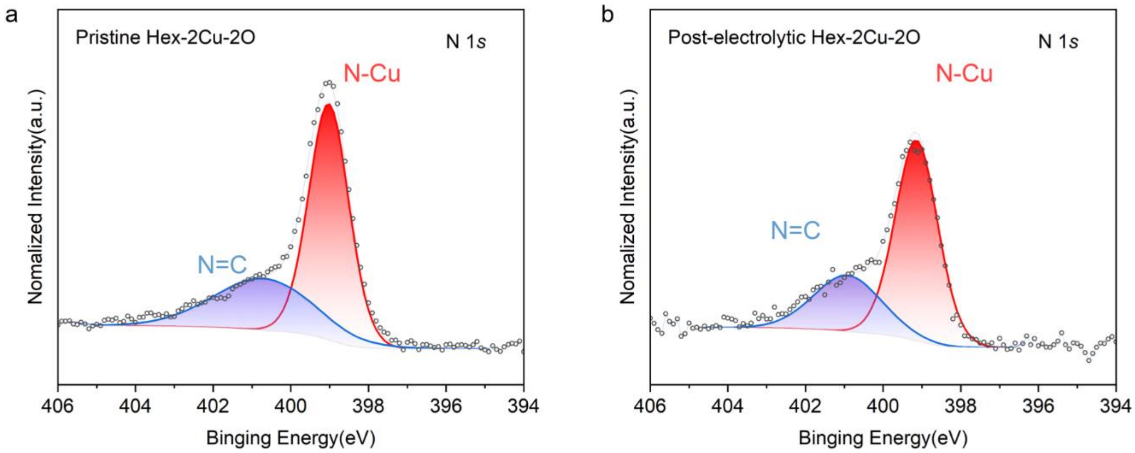

Supplementary Fig. 21 XPS N 1*s* spectra of Hex-2Cu-2O. (a) Pristine and (b) post-electrolytic Hex-2Cu-2O.

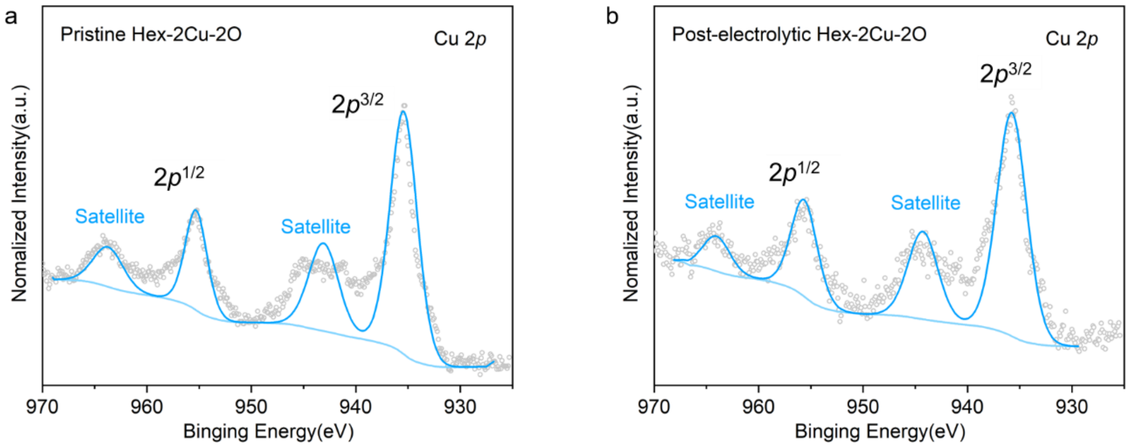

Supplementary Fig. 22 XPS Cu 2*p* spectra of Hex-2Cu-2O. (a) Pristine and (b) post-electrolytic Hex-2Cu-2O.

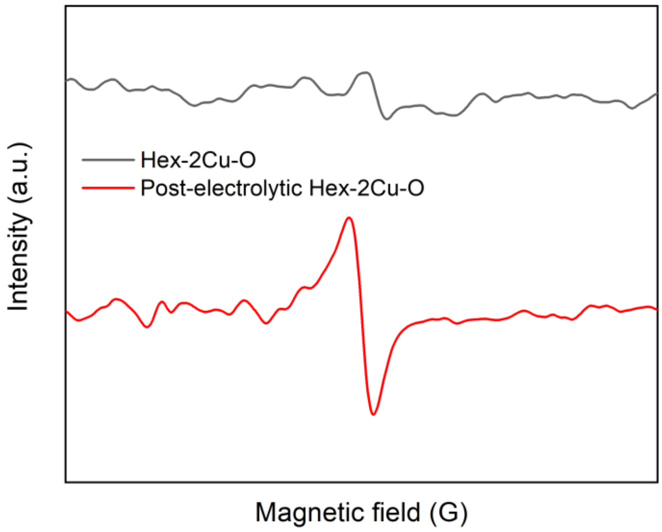

Supplementary Fig. 23 EPR spectra of Hex-2Cu-O (gray) and post-electrolytic Hex-2Cu-O (red).

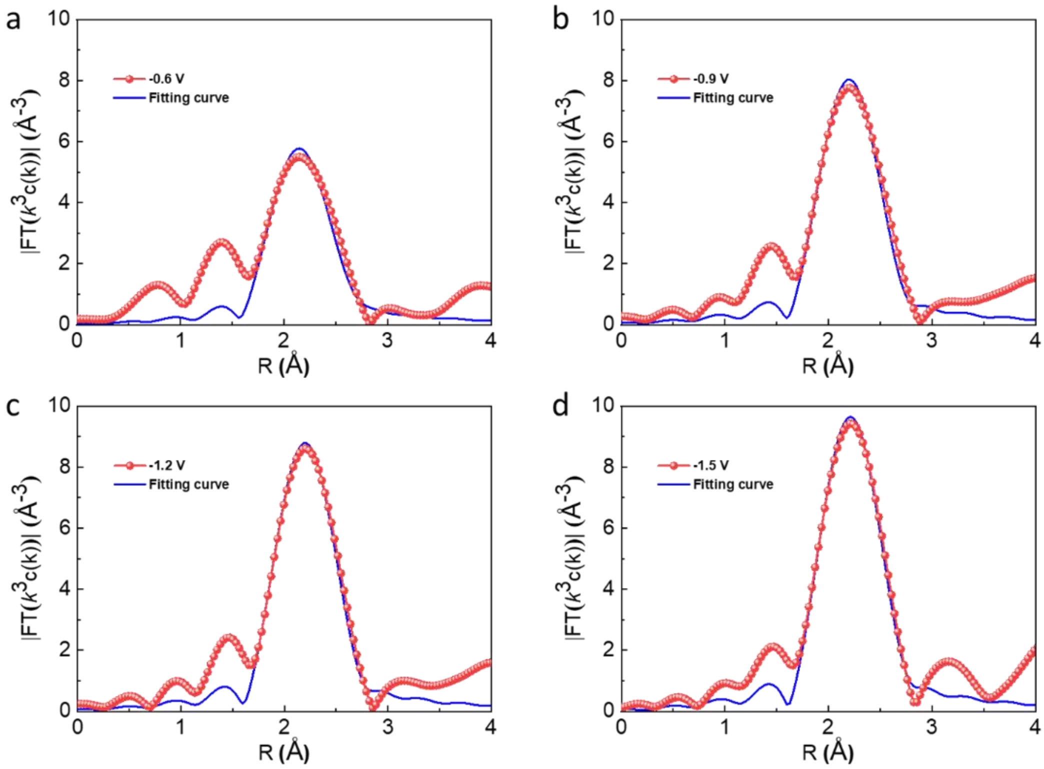

Supplementary Fig. 24 Fitting of the in-situ EXAFS Cu K-edge spectra for Hex-2Cu-O. (a) -0.6V, (b) -0.9V, (c) -1.2V and (d) -1.5V.

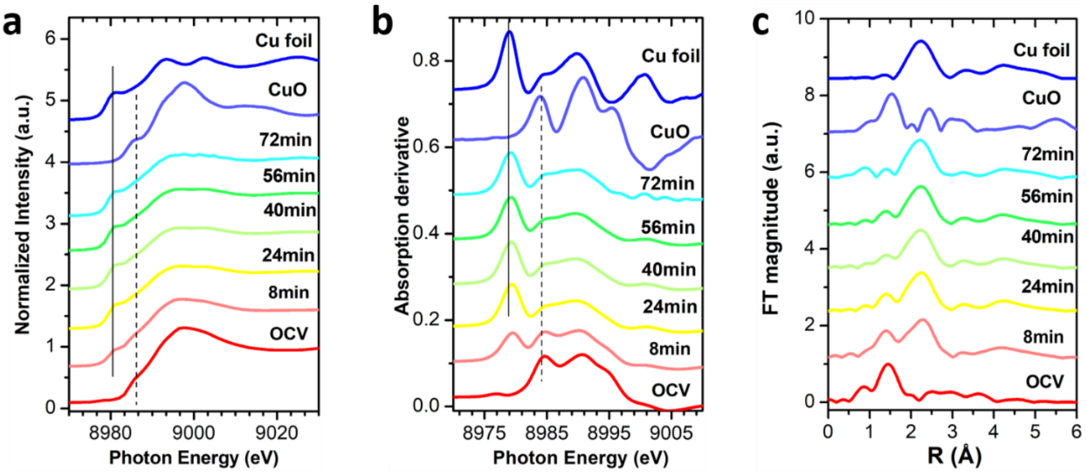

Supplementary Fig. 25 Time-lapse operando XAS measurements on Hex-2Cu-O during CO_2RR at -1.2 V vs RHE. (a) Cu K-edge XANES spectra; (b) first-order derivatives of the XANES spectra; (c) Fourier-transform EXAFS spectra.

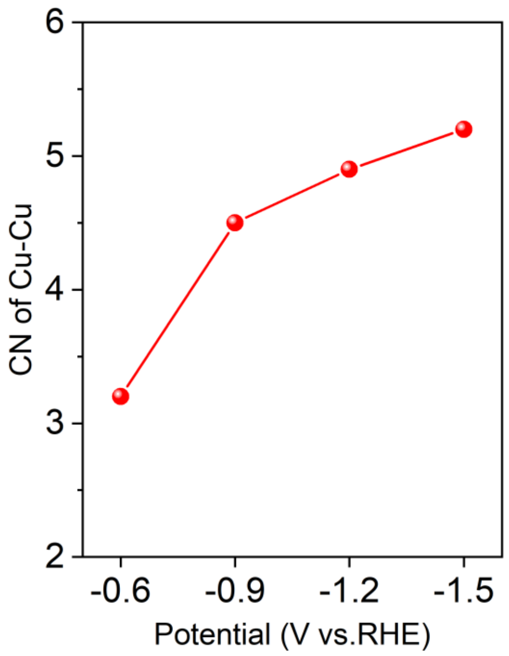

Supplementary Fig. 26 Plot of the first-shell Cu–Cu CNs as a function of applied potential for Hex-2Cu-O.

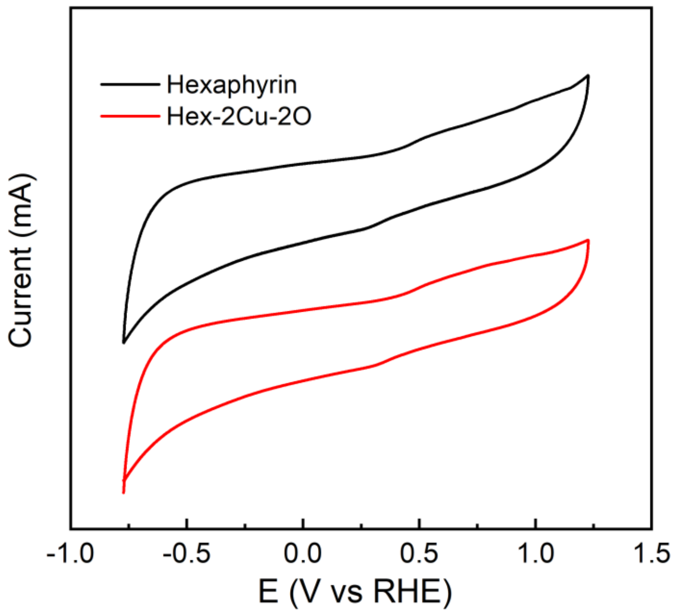

Supplementary Fig. 27 Redox properties analysis of Hex-2Cu-2O and Hexaphyrin. Cyclic voltammetry (CV) curves between -0.8 and 1.2 V at a scanning rate of 50 mV s^{-1} for Hex-2Cu-2O and Hexaphyrin in Ar-saturated 0.1 M $KHCO_3$ electrolyte.

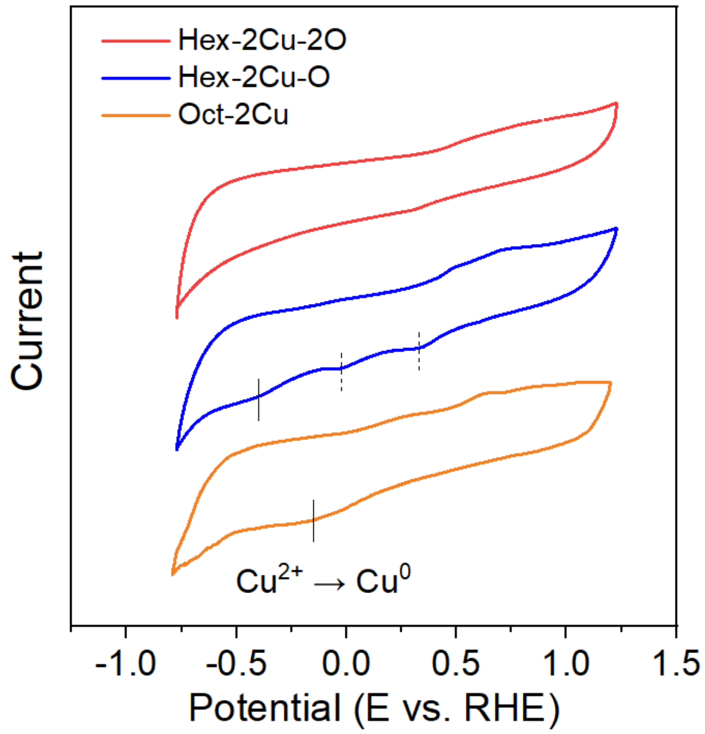

Supplementary Fig. 28 Redox properties analysis of Hex-2Cu-2O, Hex-2Cu-O and Oct-2Cu. Cyclic voltammetry (CV) curves between -0.8 and 1.2 V at a scanning rate of 50 mV s^{-1} for Hex-2Cu-2O, Hex-2Cu-O and Oct-2Cu in Ar-saturated 0.1 M $KHCO_3$ electrolyte.

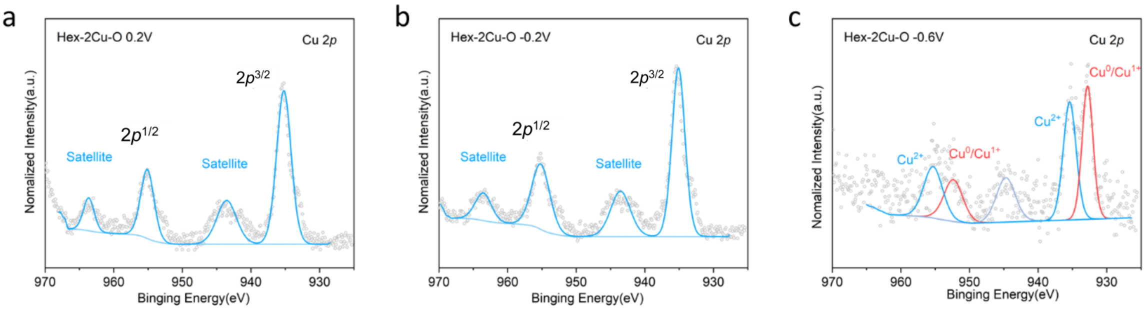

Supplementary Fig. 29 XPS analysis of post-electrolytic Hex-2Cu-O. XPS Cu 2*p* spectra taken on post-electrolytic Hex-2Cu-O after bulk analysis at (a) 0.2, (b) -0.2 and (c) -0.6 V in Ar-saturated 0.1 M $KHCO_3$ electrolyte.

a

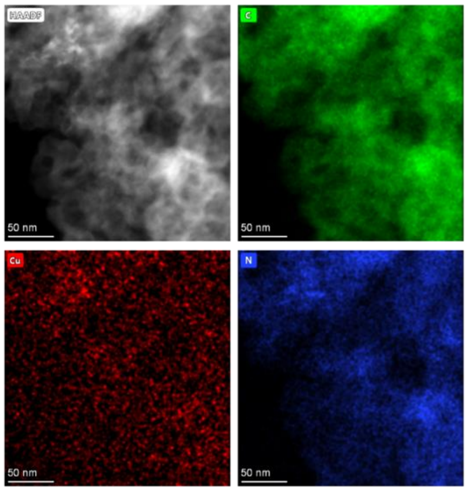

b

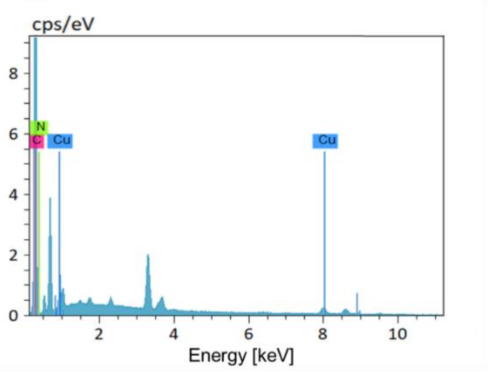

c

Z	Element	Family	Quality (%)	Atomic Fraction (%)	Atomic Error (%)
6	C	K	80.00	89.39	3.81
7	N	K	8.54	8.19	0.49
29	Cu	K	11.46	2.42	0.31

Supplementary Fig. 30 TEM image and corresponding element analysis of Hex-2Cu-O electrode before ethanol soaking. (a) EDX mapping images, (b) EDX spectrum and (c) the corresponding elemental analysis of the post-electrolytic Hex-2Cu-O electrode before ethanol soaking.

a

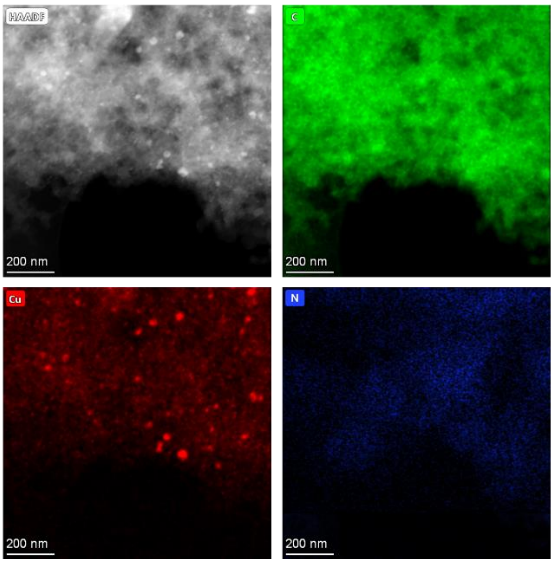

b

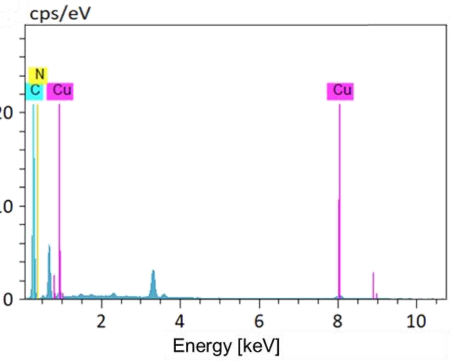

c

Z	Element	Family	Quality (%)	Atomic Fraction (%)	Atomic Error (%)
6	C	K	86.60	94.29	4.07
7	N	K	4.05	3.79	0.25
29	Cu	K	9.35	1.92	0.26

Supplementary Fig. 31 TEM image and corresponding element analysis of Hex-2Cu-O electrode after ethanol soaking. (a) EDX mapping images, (b) EDX spectrum and (c) the corresponding elemental analysis of the post-electrolytic Hex-2Cu-O electrode after ethanol soaking.

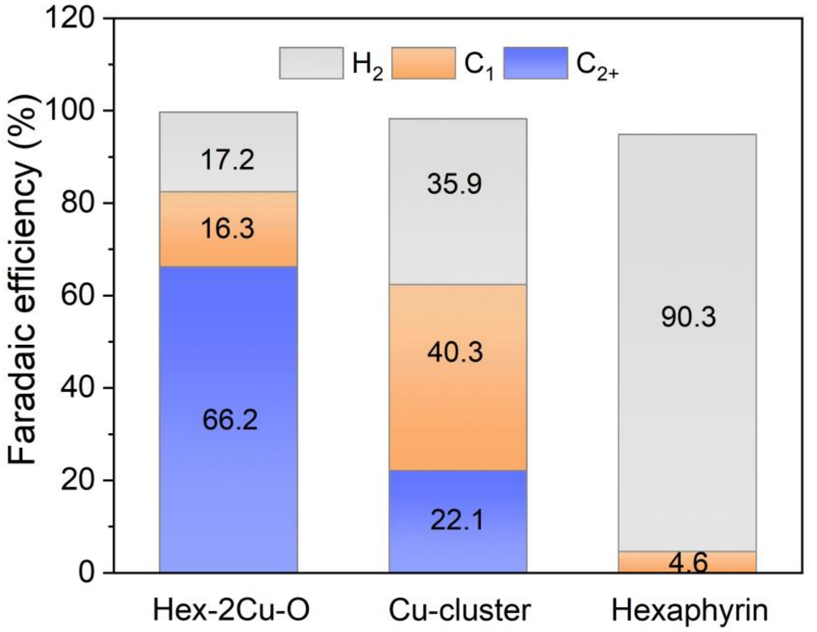

Supplementary Fig.32 CO_2RR product distribution and the corresponding FEs for Hex-2Cu-O, post-electrolytic Hex-2Cu-O after ethanol soaking (labeled as Cu-cluster), and the freebase hexaphyrin at -1.2 V vs RHE.

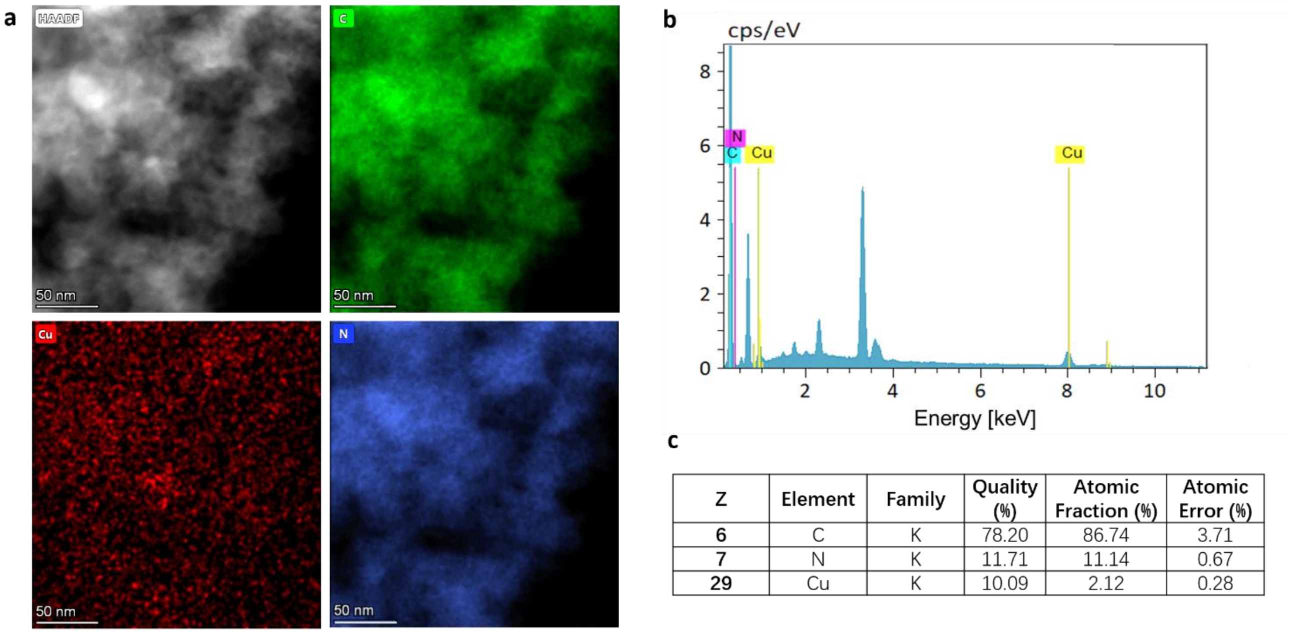

Z	Element	Family	Quality (%)	Atomic Fraction (%)	Atomic Error (%)
**6**	C	K	78.20	86.74	3.71
**7**	N	K	11.71	11.14	0.67
**29**	Cu	K	10.09	2.12	0.28

Supplementary Fig. 33 TEM image and corresponding element analysis of Hex-2Cu-O electrode after casting back the soaking solution. (a) EDX mapping images, (b) EDX spectrum and (c) the corresponding elemental analysis of the post-electrolytic Hex-2Cu-O electrode after casting back the solute from the soaking solution.

	**Hex-2Cu-O**
**Models after geometry optimization**	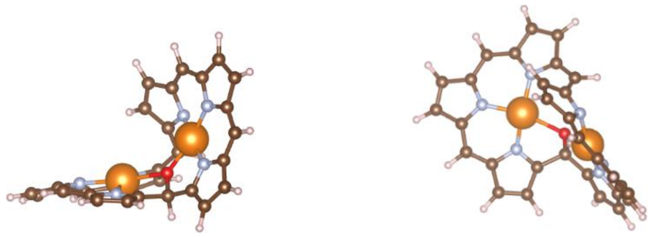
**Models after electrolysis**	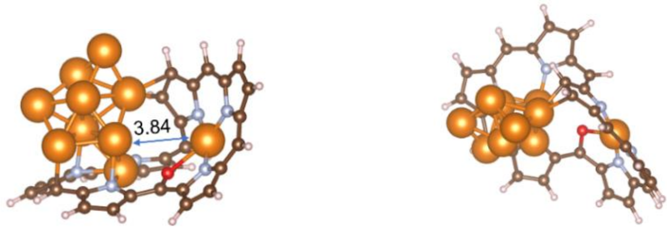 R-Hex-2Cu-O (partially reduced)
**Models with graphene for mechanism study**	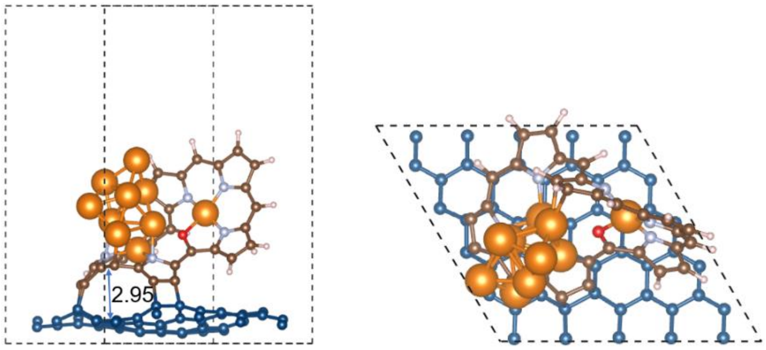 R-Hex-2Cu-O/G

Supplementary Fig. 34 Atomic structures of Hex-2Cu-O. The upper and lower panel are front and top views, respectively. Colour codes: Cu, orange; C, brown (Hex-2Cu-O) and navy (graphene); O, red; N, silver and H, pink. The distance is marked in Å.

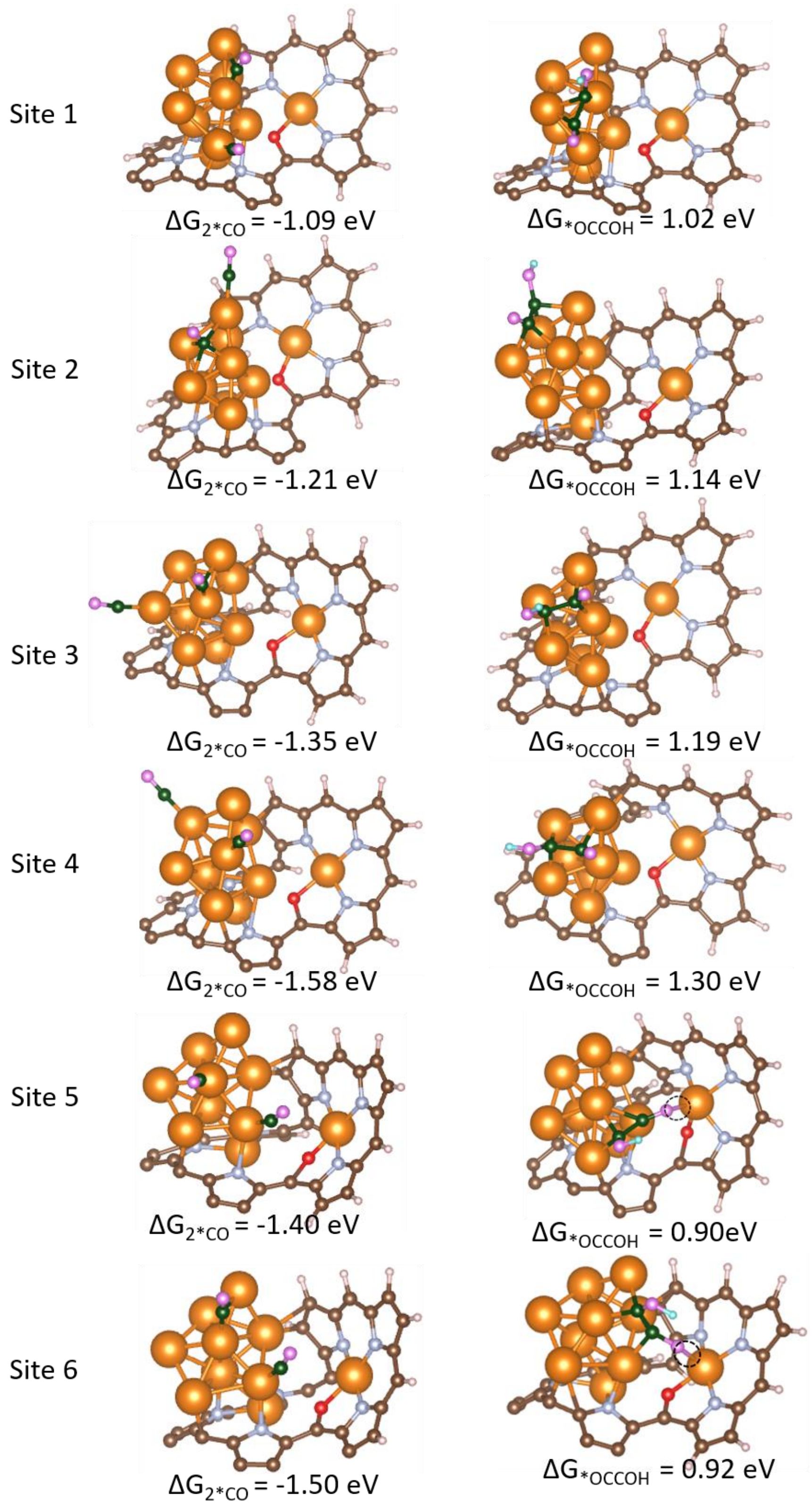

Supplementary Fig. 35 Active sites identified for C–C coupling on R-Hex-2Cu-O/G and the relevant adsorption energies. Colour codes: Cu, orange; C, brown (Hex-2Cu-O) and green (reaction intermediates); O, red (Hex-2Cu-O) and purple (reaction intermediates); N, silver and H, pink (Hex-2Cu-O) and blue (reaction intermediates). The graphene substrates were not displayed for visual convenience, and the extra O–Cu bonds were highlighted with black dashed circles.

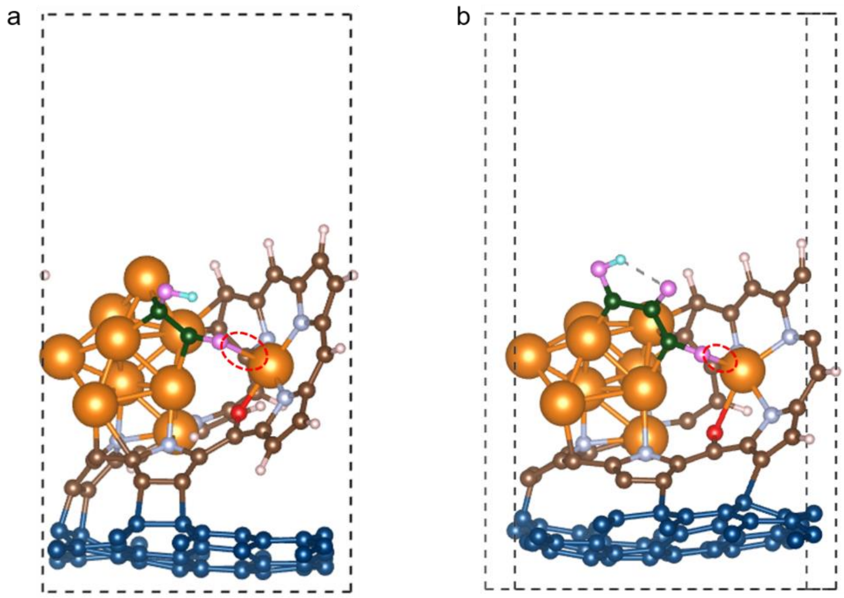

Supplementary Fig. 36 Atomic structure of intermediates on R-Hex-2Cu-O/G. (a) *OCCOH and (b) *OCCOCOH adsorbed on R-Hex-2Cu-O/G. Due to the confined space, both intermediates have an extra bonding with adjacent single Cu center, which are highlighted with red dashed circles. Colour codes: Cu, orange; C, brown (Hex-2Cu-O), navy (graphene) and green (reaction intermediates); O, red (Hex-2Cu-O) and purple (reaction intermediates); N, silver and H, pink (Hex-2Cu-O) and blue (reaction intermediates).

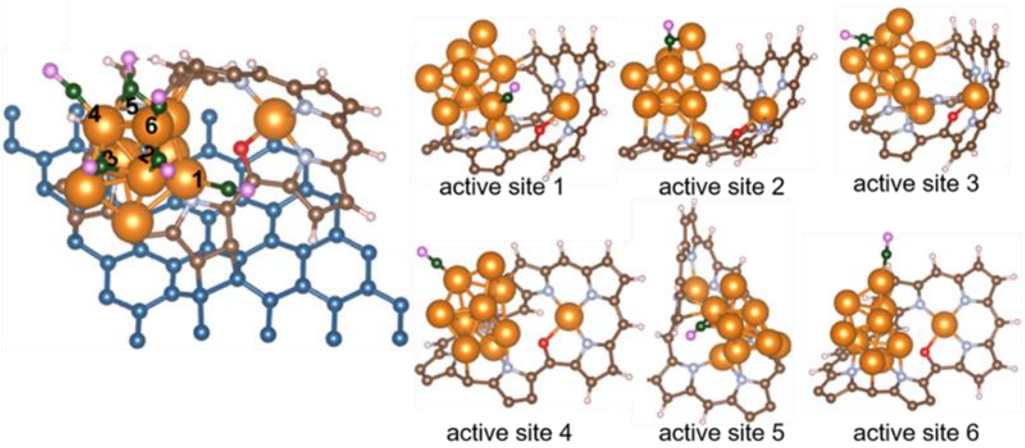

Supplementary Fig. 37 *CO and *H adsorption sites identified on R-Hex-2Cu-O/G surface. The left panel is an overall view of active sites, and right panel is the detailed picture of each active site with graphene substrates not displayed for visual convenience. *CO was used as the exemplified atomic configuration. Colour codes: Cu, orange; C, brown (Hex-2Cu-O), green (reaction intermediates) and navy (graphene); O, red (Hex-2Cu-O) and purple (reaction intermediates); N, silver and H, pink.

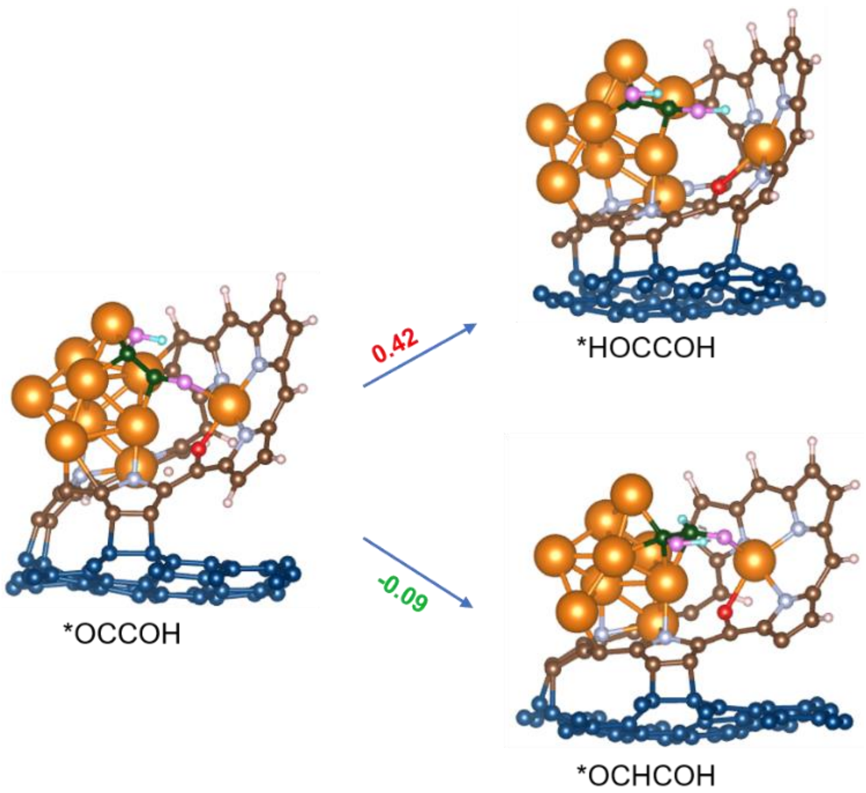

Supplementary Fig. 38 *OCCOH protonation to *HOCCOH vs *OCHCOH on R-Hex-2Cu-O/G. Free energy change at 0 V-RHE (ΔG) values appear in eV. Green and red values denote, respectively, the exergonic and endergonic process. Colour codes: Cu, orange; C, brown (Hex-2Cu-O), navy (graphene) and green (reaction intermediates); O, red (Hex-2Cu-O) and purple (reaction intermediates); N, silver and H, pink (Hex-2Cu-O) and blue (reaction intermediates).

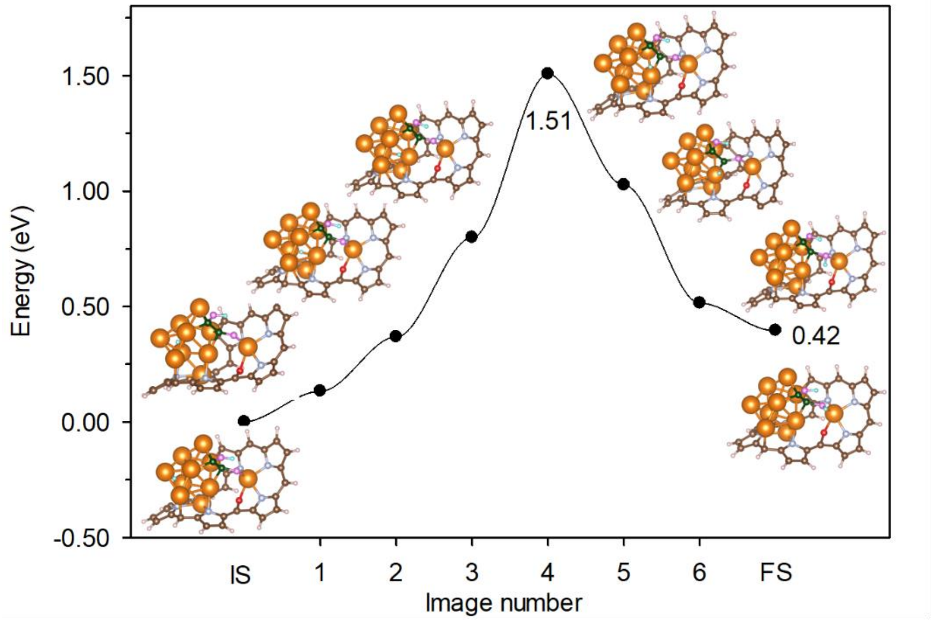

Supplementary Fig. 39 Minimum energy path (MEP) involved in *OCCOH + *H → *HOCCOH with R-Hex-2Cu-O/G at 0 V-RHE. The reference energy level is set to be adsorption of initial state. Insets are atomistic structures of the initial, transition and final states. Colour codes: Cu, orange; C, brown (Hex-2Cu-O), and green (reaction intermediates); O, red (Hex-2Cu-O) and purple (reaction intermediates); N, silver and H, pink (Hex-2Cu-O) and blue (reaction intermediates).

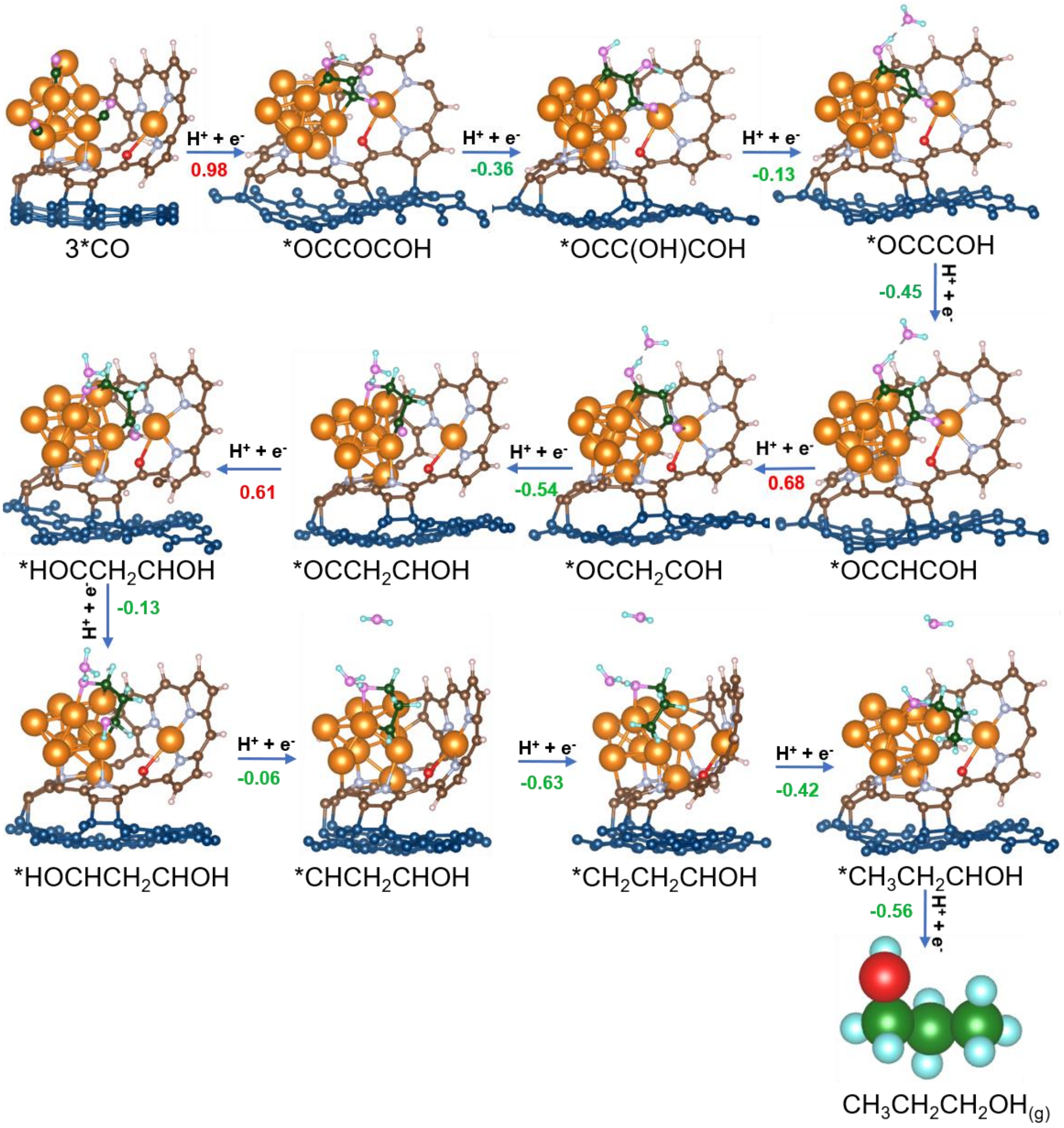

Supplementary Fig. 40 Atomic structures of the reaction intermediates along the pathway of reduction 3*CO to n-propanol on R-Hex-2Cu-O/G. Free energy change at 0 V vs RHE (ΔG) values appear in eV. Green and red values denote, respectively, exergonic and endergonic process. Colour codes: Cu, orange; C, brown (Hex-2Cu-O), navy (graphene) and green (reaction intermediates); O, red (Hex-2Cu-O) and purple (reaction intermediates); N, silver and H, pink (Hex-2Cu-O) and blue (reaction intermediates).

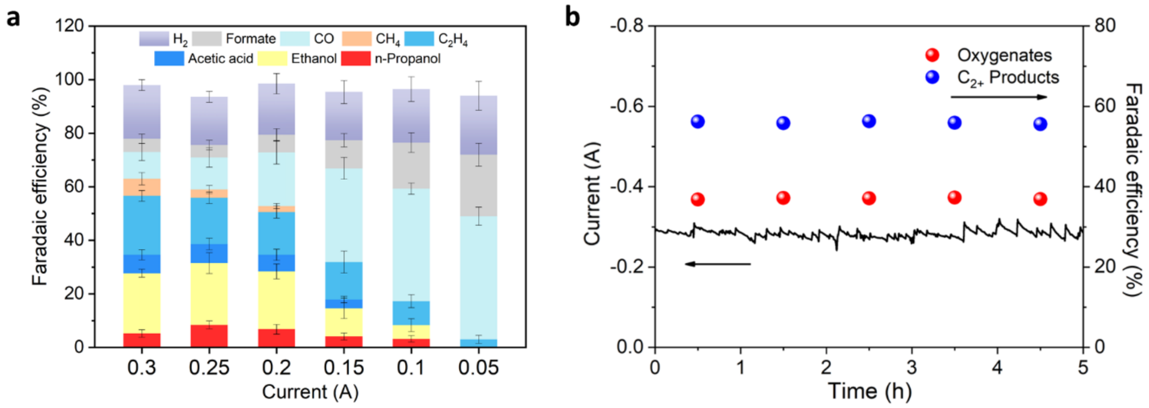

Supplementary Fig. 41 Electrocatalytic CO_2RR performances of Hex-2Cu-O in a flow-cell with 1 M KOH electrolyte. (a) Current-dependent FEs of various reduction products. (b) Chronoamperometric i–t curve along with the FEs of oxygenates and C_{2+} products (right axis) at −0.66 V (vs RHE with IR correction) for a testing period of 5 h. Error bars represent the standard deviation of three independent measurements.

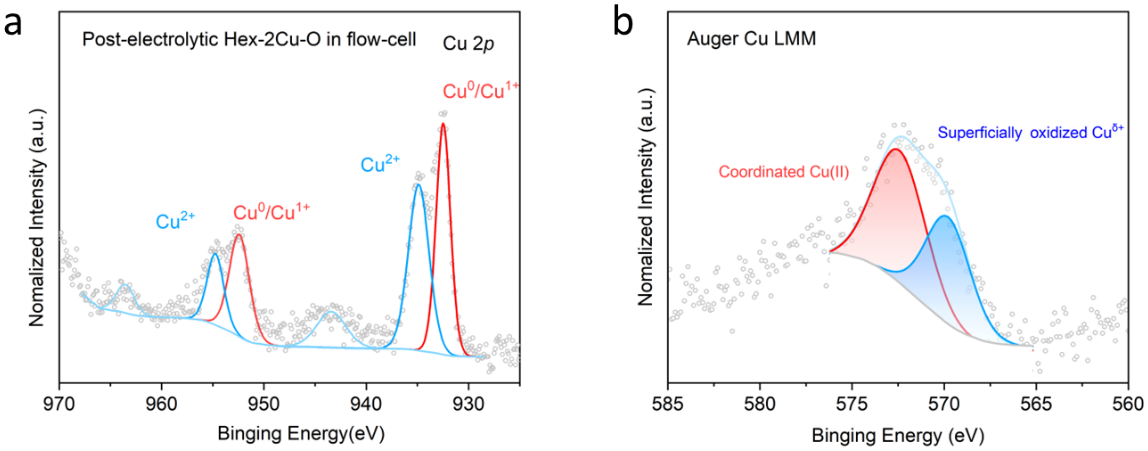

Supplementary Fig. 42 XPS analysis of Hex-2Cu-O after the chronoamperometric i-t test in flow cell. (a) XPS Cu 2*p* and (b) Auger Cu LMM spectra.

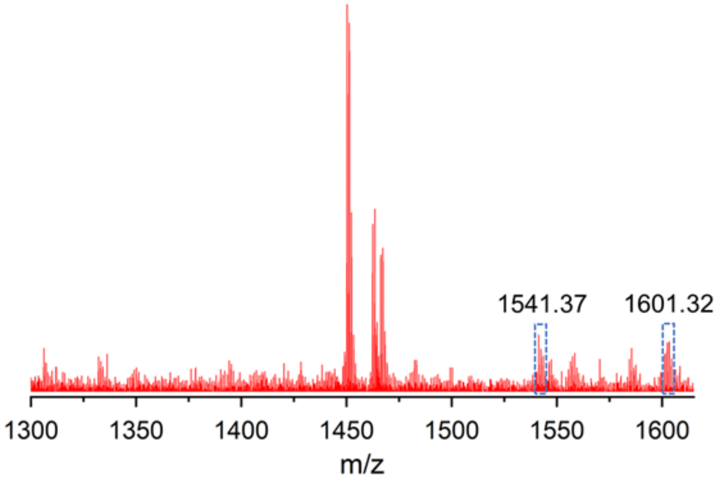

Supplementary Fig. 43 MALDI-TOF MS spectrum of the organic phase of post-electrolytic Hex-2Cu-O after the chronoamperometric i-t test in flow cell.

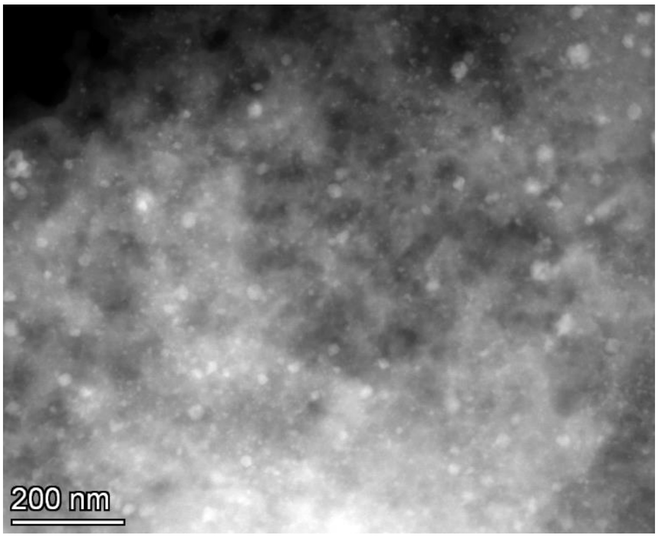

Supplementary Fig. 44 TEM image of post-electrolytic Hex-2Cu-O after the chronoamperometric i-t test in flow cell.

Supplementary Table 1. Bond length data in the first-shell Cu coordination for the three bicentric complexes[1,2].

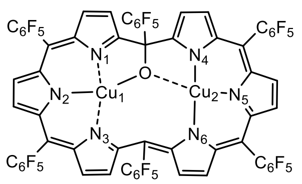

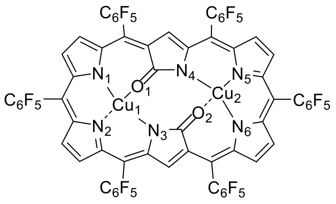

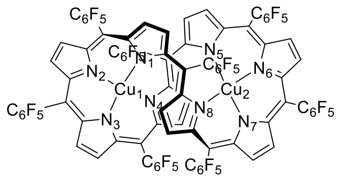

Hex-2Cu-O		Hex-2Cu-2O		Oct-2Cu	
**Bond**	**Length (Å)**	**Bond**	**Length (Å)**	**Bond**	**Length (Å)**
**Cu_1-N_1**	1.87	**Cu_1-N_1**	2.04	**Cu_1-N_1**	2.06
**Cu_1-N_2**	1.96	**Cu_1-N_2**	1.96	**Cu_1-N_2**	1.97
**Cu_1-N_3**	1.91	**Cu_1-N_3**	2.05	**Cu_1-N_3**	1.98
**Cu_1-O**	2.05	**Cu_1-O_1**	1.93	**Cu_1-N_4**	2.02
**Cu_2-O**	2.15	**Cu_2-O_2**	1.93	**Cu_2-N_5**	2.06
**Cu_2-N_4**	1.90	**Cu_2-N_4**	2.05	**Cu_2-N_6**	1.97
**Cu_2-N_5**	1.99	**Cu_2-N_5**	1.96	**Cu_2-N_7**	1.98
**Cu_2-N_6**	1.93	**Cu_2-N_6**	2.04	**Cu_2-N_8**	2.02

Supplementary Table 2. Comparison of C_{2+} alcohols production on various Cu-based catalysts conducted at room temperature.

Catalysts	Electrolyte	Potential (V vs. RHE)	Faradaic efficiency (FE)	Partial Current density (mA cm^{-2})	References
**Hex-2Cu-O**	**1 M KOH**	**-0.66 V**	**31.5% (Ethanol+n-PrOH in Flow-Cell)**	**87**	**this work**
**Hex-2Cu-O**	**0.1 M $KHCO_3$**	**-1.2 V**	**53.1% (Ethanol+n-PrOH in H-Cell)**	**6.5**	**this work**
**Nanoporous Ag/Cu**	1 M KOH	–0.7 V	25% (Ethanol)	80	Ref.3
**Binding-site diverse Ag/Cu**	1 M KOH	–0.67 V	~41% (Ethanol)	102.5	Ref.4
**CuS_x-DSV**	0.1 M $KHCO_3$	–0.92 V	~15.1% (PrOH)	9.9	Ref.5
**Cu_2-CuN_3**	0.1 M $KHCO_3$	-1.2 V	51% (Ethanol)	14.4	Ref.6
**Cu clusters on Oxidized Carbon**	0.1 M $KHCO_3$	–0.7 V	91% (Ethanol)	1.5	Ref.7
**Cu particles**	2 M KCl	-1.2V	31.1%(Ethanol)	31.1	Ref.8

Supplementary Table 3. ^{1}H-NMR data points from the stability test.

Time (hours)	Products(mmol/L)		
	Formate	Ethanol	n-Propanol
2	0.265	0.0715	0.0608
4	0.432	0.188	0.0937
6	0.59	0.305	0.126
8	0.76	0.422	0.159
10	0.93	0.539	0.192
12	1.10	0.656	0.224
14	1.26	0.773	0.257
16	1.43	0.890	0.290
18	1.60	1.00	0.323
20	1.77	1.12	0.356
22	1.93	1.24	0.389
24	2.10	1.35	0.421

Supplementary Table 4. Structure parameters extracted from the EXAFS fitting of Cu K-edge.

**Sample**	**Path**	S_0^2	**CN**	**R(Å)**	ΔE_0(eV)	$\sigma^2(10^{-3}$ Å$^2)$
Hex-2Cu-O-0.6 V	Cu-Cu	0.88	3.2	2.53	-7.4	8.67
Hex-2Cu-O-0.9 V	Cu-Cu	0.88	4.5	2.52	3.0	9.17
Hex-2Cu-O-1.2 V	Cu-Cu	0.88	4.9	2.53	2.7	9.27
Hex-2Cu-O-1.5 V	Cu-Cu	0.88	5.2	2.53	3.5	8.81

[a]CN is the coordination number; R is interatomic distance (the bond length between central atoms and surrounding coordination atoms); ΔE_0 is edge-energy shift (the difference between the zero kinetic energy value of the sample and that of the theoretical model); σ^2 is Debye-Waller factor (a measure of thermal and static disorder in absorber-scatterer distances).

Supplementary Table 5. EDX elemental analysis of copper content on the Hex-2Cu-O electrode before and after ethanol soaking, as well as after casting back the solute from the soaking solution.

**Post-mortem Treatments**	**Cu (%)**	**N (%)**
Before soaking	11.46	8.54
After soaking	9.35	4.05
After casting back the solute from the soaking solution	10.09	11.71

Supplementary Table 6. Thermodynamic profile regarding all PCET steps for reduction 2*CO to ethanol on R-Hex-2Cu-O/G. The energy unit is eV. The favourable species are colored in black while less favorable species are displayed in red.

PCET step	Reaction	Δ*G*
5th	$*CO + *CO + H^+ + e^- \rightarrow *OCCOH$	0.92
6th	$*OCCOH + H^+ + e^- \rightarrow *OCHCOH$	–0.09
	$*OCCOH + H^+ + e^- \rightarrow *HOCCOH$	0.42 (E_a = 1.51)
	$*OCCOH + H^+ + e^- \rightarrow *CCO + H_2O$	Not stable
7th	$*OCHCOH + H^+ + e^- \rightarrow *OCHC + H_2O$	–1.19
	$*OCHCOH + H^+ + e^- \rightarrow *OCHCHOH$	–0.25
8th	$*OCHC + H^+ + e^- \rightarrow *OCHCH$	–0.07
	$*OCHC + H^+ + e^- \rightarrow *CCHOH$	0.05
9th	$*OCHCH + H^+ + e^- \rightarrow *OCHCH_2$	–0.03
	$*OCHCH + H^+ + e^- \rightarrow *CHCHOH$	0.17
10th	$*OCHCH_2 + H^+ + e^- \rightarrow *OCHCH_3$	–0.71
	$*OCHCH_2 + H^+ + e^- \rightarrow *OCH_2CH_2$	0.39
11th	$*OCHCH_3 + H^+ + e^- \rightarrow *OCH_2CH_3$	–0.03
12th	$*OCH_2CH_3 + H^+ + e^- \rightarrow CH_3CH_2OH(g)$	0.49

Supplementary Table 7. Thermodynamic profile regarding all PCET steps for reduction 2*CO to ethylene on R-Hex-2Cu-O/G. The energy unit is eV.

PCET step	Reaction	ΔG
5th	$*CO + *CO + H^+ + e^- \rightarrow *OCCOH$	0.92
6th	$*OCCOH + H^+ + e^- \rightarrow *HOCCOH$	0.42
7th	$*HOCCOH + H^+ + e^- \rightarrow *CCOH + H_2O$	0.54
8th	$*CCOH + H_2O + H^+ + e^- \rightarrow *CHCOH + H_2O$	–1.11
9th	$*CHCOH + H_2O + H^+ + e^- \rightarrow *CCH + 2H_2O$	–0.34
10th	$*CCH + 2H_2O + H^+ + e^- \rightarrow *CCH_2 + 2H_2O$	–0.72
11th	$*CCH_2 + 2H_2O + H^+ + e^- \rightarrow *CHCH_2 + 2H_2O$	–0.05
12th	$*CHCH_2 + 2H_2O + H^+ + e^- \rightarrow CH_2CH_2(g) + 2H_2O$	–0.06

Supplementary Table 8. Thermodynamic profile regarding all PCET steps for reduction 3*CO to n-propanol on R-Hex-2Cu-O/G. The energy unit is eV. The favourable species are colored in black while less favorable species are displayed in red.

PCET step	Reaction	ΔG
5th	*CO + *CO + *CO + H^+ + e^- → *OCCOCOH	0.98
6th	*OCCOCOH + H^+ + e^- → *OCC(OH)COH	–0.36
7th	*OCC(OH)COH + H^+ + e^- → *OCCCOH + H_2O	–0.13
8th	*OCCCOH + H^+ + e^- → *OCCHCOH	–0.45
9th	*OCCHCOH + H^+ + e^- → *OCCH_2COH	0.68
	*OCCHCOH + H^+ + e^- → *CCHCO + H_2O	1.10
	*OCCHCOH + H^+ + e^- → *C(OH)CHCOH	0.91
10th	*OCCH_2COH + H^+ + e^- → *OCCH_2CHOH	–0.54
	*OCCH_2COH + H^+ + e^- → *OCCH_2C + H_2O	0.58
11th	*OCCH_2CHOH + H^+ + e^- → *HOCCH_2CHOH	0.61
	*OCCH_2CHOH + H^+ + e^- → *OCCH_2CH + H_2O	0.86
12th	*HOCCH_2CHOH + H^+ + e^- → *HOCHCH_2CHOH	–0.13
	*HOCCH_2CHOH + H^+ + e^- → *CCH_2CHOH + H_2O	0.34
13th	*HOCHCH_2CHOH + H^+ + e^- → *CHCH_2CHOH + H_2O	–0.06
14th	*CHCH_2CHOH + H^+ + e^- → *CH_2CH_2CHOH	–0.63
15th	*CH_2CH_2CHOH + H^+ + e^- → *CH_3CH_2CHOH	–0.42
16th	*CH_3CH_2CHOH + H^+ + e^- → $CH_3CH_2CH_2OH_{(g)}$	–0.56

Supplementary Table 9. Free energies for gas phase species.

Gas Molecule	E (eV)	ZPE (eV)	-TS (eV)	G (eV)
**H_2**	-6.75	0.27	-0.41	-6.89
**H_2O**	-14.22	0.56	-0.67	-14.33
**CO**	-14.80	0.13	-0.61	-15.28
**CO_2**	-23.01	0.31	-0.66	-23.36
**C_2H_4**	-31.97	1.37	-0.55	-31.15
**C_2H_5OH**	-46.88	2.13	-0.60	-45.35

Supplementary Table 10. Vibrational frequency of transition states identified in this research.

***OCCOH + *H → *HOCCOH**
1 f = 3494.502529 cm^{-1}
2 f = 1382.272709 cm^{-1}
3 f = 1263.112144 cm^{-1}
4 f = 1253.379407 cm^{-1}
5 f = 1164.030598 cm^{-1}
6 f = 957.173966 cm^{-1}
7 f = 942.763338 cm^{-1}
8 f = 766.486699 cm^{-1}
9 f = 614.822362 cm^{-1}
10 f = 490.744673 cm^{-1}
11 f = 378.359316 cm^{-1}
12 f = 277.744142 cm^{-1}
13 f = 236.367804 cm^{-1}
14 f = 227.029943 cm^{-1}
15 f = 152.336569 cm^{-1}
16 f = 104.395047 cm^{-1}
17 f = 65.490684 cm^{-1}
18 f/i= 478.271258 cm^{-1}

References:

1 Tanaka, Y. *et al.* Thermal Splitting of Bis-Cu(II) Octaphyrin(1.1.1.1.1.1.1.1) into Two Cu(II) Porphyrins. *J. Am. Chem. Soc.* **126**, 3046-3047 (2004).

2 Shimizu, S. *et al.* Biscopper Complexes of Meso-Aryl-Substituted Hexaphyrin: Gable Structures and Varying Antiferromagnetic Coupling. *J. Am. Chem. Soc.* **126**, 12280-12281 (2004).

3 Hoang, T. T. H. *et al.* Nanoporous Copper-Silver Alloys by Additive-Controlled Electrodeposition for the Selective Electroreduction of CO_2 to Ethylene and Ethanol. *J Am Chem Soc* **140**, 5791-5797 (2018).

4 Li, Y. C. *et al.* Binding Site Diversity Promotes CO_2 Electroreduction to Ethanol. *J Am Chem Soc* **141**, 8584-8591 (2019).

5 Peng, C. *et al.* Double Sulfur Vacancies by Lithium Tuning Enhance CO_2 Electroreduction to N-Propanol. *Nat. Commun.* **12**, 1580 (2021).

6 Su, X. *et al.* Complementary Operando Spectroscopy Identification of in-Situ Generated Metastable Charge-Asymmetry Cu_2-CuN_3 Clusters for CO_2 Reduction to Ethanol. *Nat. Commun.* **13**, 1322 (2022).

7 Xu, H. *et al.* Highly Selective Electrocatalytic CO_2 Reduction to Ethanol by Metallic Clusters Dynamically Formed from Atomically Dispersed Copper. *Nat. Energy* **5**, 623-632 (2020).

8 Zhang, X. *et al.* Selective and High Current CO_2 Electro-Reduction to Multicarbon Products in near-Neutral Kcl Electrolytes. *J Am Chem Soc* **143**, 3245-3255 (2021).
